# Propofol Inhibits Ischemia/Reperfusion-Induced Cardiotoxicity Through the Protein Kinase C/Nuclear Factor Erythroid 2-Related Factor Pathway

**DOI:** 10.3389/fphar.2021.655726

**Published:** 2021-05-13

**Authors:** Shengqiang Li, Zhen Lei, Meng Zhao, Yonghao Hou, Di Wang, Xingli Xu, Xiaowen Lin, Jingxin Li, Shuhai Tang, Jingui Yu, Tao Meng

**Affiliations:** ^1^ Department of Anesthesiology, Qilu Hospital, Cheeloo College of Medicine, Shandong University, Jinan, China; ^2^ Department of Anesthesiology, The Second Hospital, Cheeloo College of Medicine, Shandong University, Jinan, China; ^3^ Department of Cardiovascular Ultrasound and Non-invasive Cardiology, Sichuan People’s Hospital, Chengdu, China; ^4^ Department of Pain Management, Shandong Provincial Hospital Affiliated to Shandong First Medical University, Jinan, China; ^5^ Department of Physiology, Cheeloo College of Medicine, Shandong University, Jinan, China

**Keywords:** propofol, heart, ischemia reperfusion, protein kinase C, nuclear factor erythroid-2- related factor, heme oxygenase-1

## Abstract

Both hydrogen peroxide (H_2_O_2_, H) and ischemia/reperfusion (I/R) can damage cardiomyocytes, which was inhibited by propofol (P). The present research was designed to examine whether propofol can reduce myocardial I/R injury by activating protein kinase C (PKC)/nuclear factor erythroid-2-related factor 2 (NRF2) pathway in H9C2 cells and rat Langendorff models. H9C2 cells were disposed of no reagents (C), H_2_O_2_ for 24 h (H), propofol for 1 h before H_2_O_2_ (H+P), and chelerythrine (CHE, PKC inhibitor) for 1 h before propofol and H_2_O_2_ (H+P+CHE). N = 3. The PKC gene of H9C2 was knocked down by siRNA and overexpressed by phorbol 12-myristate 13-acetate (PMA, PKC agonist). The cell viability and the expressions of PKC, NRF2, or heme oxygenase-1(HO-1) were evaluated. Propofol significantly reduced H9C2 cell mortality induced by H_2_O_2_, and significantly increased NRF2 nuclear location and HO-1 expression, which were restrained by siRNA knockout of PKC and promoted by PMA. Rat hearts were treated with KrebsHenseleit solution for 120 min (C), with (I/R+P) or without (I/R) propofol for 20 min before stopping perfusion for 30 min and reperfusion for 60 min, and CHE for 10 min before treated with propofol. N = 6. The levels of lactate dehydrogenase (LDH), superoxide dismutase (SOD), and creatine kinase-MB (CK-MB) in perfusion fluid and antioxidant enzymes in the myocardium were assessed. I/R, which increased LDH and CK-MB expression and reduced SOD expression, boosted the pathological damage and infarcts of the myocardium after reperfusion. However, propofol restrained all these effects, an activity that was antagonized by CHE. The results suggest that propofol pretreatment protects against I/R injury by activating of PKC/NRF2 pathway.

## Introduction

Acute myocardial infarction (AMI) is one of the leading causes of death and disability globally (Zhou et al., 2018). The most effective treatment is early myocardial reperfusion (Zhao et al., 2018), which can also aggravate myocardial damage (Majidi et al., 2009) due to ischemia/reperfusion (I/R) injury (Tian et al., 2017; Zhang et al., 2019). I/R injury is probably caused by the reactive oxygen species (ROS) overproduction, calcium overload, apoptosis in cardiomyocytes (Hoffman et al., 2004), and inflammatory response activation (Bartekova et al., 2018). Currently, symptomatic treatment is still the major treatment (Zhao et al., 2013). Therefore, protecting the myocardium from I/R injury is critically important in myocardial protection.

As an intravenous anesthetic, propofol (2, 6-diisopropyl phenol) can suppress myocardial dysfunction and reduce the infarction area after ischemia (Kobayashi et al., 2008). However, the underlying mechanism has not been elucidated. It has been hypothesized that propofol-induced myocardial protection occurs through anti-lipid peroxidation, elimination of ROS, or alleviating calcium overload (Park et al., 1995). Zhang and colleagues show that propofol reduces inflammatory cytokine and myocardial apoptosis by suppressing the Janus kinase (JAK)/signal transducer and activator of transcription (STAT) pathway (Zhang et al., 2019). Li et al. find that propofol reduces myocardial apoptosis by mediating microRNA-451/HMGB1 (Li et al., 2019). Protein kinase C (PKC), a phospholipid-dependent serine/threonine kinase, is widely expressed in the cardiovascular system. The change in PKC expression and activity represents an important biological process in the occurrence and development of many cardiovascular diseases such as heart failure, atherosclerosis, or hypertension (Budas et al., 2007; Steinberg, 2012). PKC reduces myocardial I/R injury and ameliorates heart failure (Ji et al., 2020). Propofol activates PKC isoforms in adult rat ventricular myocytes (Wickley et al., 2006) and protects the myocardium from I/R injury (Ko et al., 1997).

**FIGURE 1:**
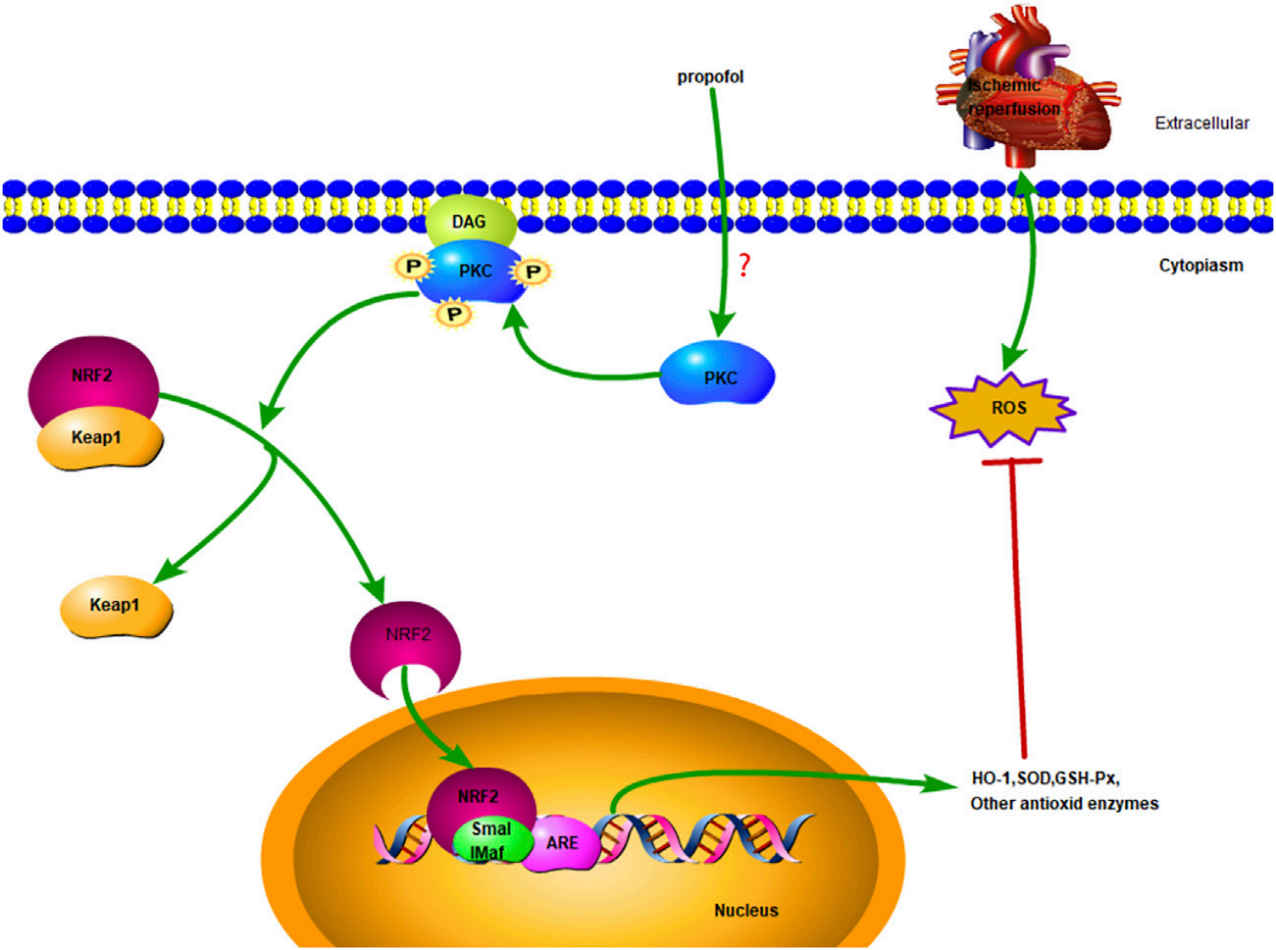
Diagrammatic sketch Propofol protects cardiomyocytes from damage caused by H_2_O_2_ or I/R oxidation through the PKC/NRF2 pathway.

The nuclear factor erythroid-2-related factor 2 (NRF2) is a central regulator of intracellular redox homeostasis (Fisher et al., 2007). Target genes of NRF2, such as heme oxygenase-1 (HO-1) or superoxide dismutase (SOD), play a protective role in the pathogenesis of cardiovascular diseases (Smith et al., 2016). PKC promotes nuclear translocation of NRF2 by activating the Ser40 phosphorylation site (Bloom and Jaiswal, 2003; Wang et al., 2020). Few studies have been investigated whether NRF2 is involved in PKC activation induced by propofol in myocardial I/R injury. The present results show that propofol induces nuclear translocation of NRF2 in the myocardium by activating PKC to prevent I/R myocardium from radical oxidation. (Figure 1).

## Materials and Methods

### Cell Culture

H9C2 cells were purchased from KeyGEN BioTECH (KG444, Nanjing, China) and grown in Dulbecco’s Modified Eagle’s Medium (DMEM, 0030034DJ, GIBCO, United States), which was supplemented with 10% fetal bovine serum (FBS, 16140071, GIBCO, United States) and 1% penicillin/streptomycin (15140122, GIBCO, United States). H9C2 cells were cultured at 37°C in a humidified atmosphere with 5% CO_2_. H9C2 cells were co-cultured with H_2_O_2_ for 24 h to establish the H_2_O_2_ oxidative damage model.

### Cell Viability Assay

The viability of H9C2 cells was evaluated using the cell counting kit-8 (CCK-8, HY-K0301, MCE, United States). Cells (3000∼4000/well) were inoculated into 96-well plates. After 24 h, different concentrations of propofol (P, 0∼800 μM, D126608, Sigma, United States), H_2_O_2_ (H, 0∼800 μM, 323381, Sigma, United States), chelerythrine (CHE, 0∼20 μM, HY-N2359, MCE, United States) or phorbol 12-myristate 13-acetate (PMA, 0∼4 μM, HY-18739, MCE, United States) were added to the cell culture. Then, 10 μl 10% CCK-8 reagent was added to each well and incubated for 1∼3 h at 37°C. The absorbance at 450 nm was measured on a Microplate Reader (Thermo Fisher Scientific, United States).

### H9C2 Cells Grouping and Corresponding Treatment

**FIGURE 2:**
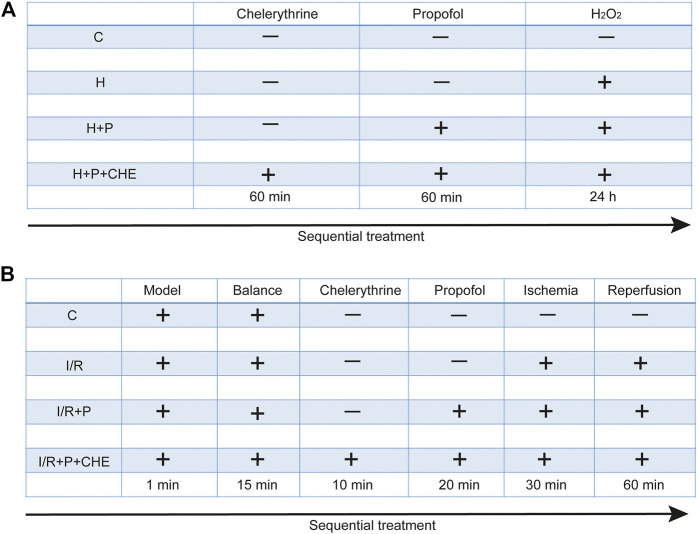
Experimental grouping and corresponding treatment on H9C2 cells **(A)** and rat heart **(B)**.

H9C2 cells were randomly assigned to four groups: 1) C: cells not treated with any reagents; 2) H: cells treated with H_2_O_2_ (200 μM) for 24 h; 3) H+P: cells treated with propofol (50 μM) for 60 min before H_2_O_2_; 4) H+P+CHE: cells treated with chelerythrine (5 μM) for 60 min before propofol and H_2_O_2_. Each experiment was replicated three times (Figure 2A).

### Trypan Blue Exclusion Assay

H9C2 cells were seeded in 12-well plates. After incubation for 24 h, the cells were treated with propofol or H_2_O_2_ as described above and then stained with 0.4% trypan blue (T8070, Solarbio life sciences, Beijing, China) at 37°C for 3 min. Cell viability was calculated on a Microplate Reader (Thermo Fisher Scientific, United States).

### Measurement of ROS Production

The ROS levels in H9C2 cells were measured using the DCFH-DA kit (S0033S Beyotime, Shanghai, China). After treatment as described above, the cells were incubated in DCFH-DA (10 μM) at 37°C for 1 h. Excitation at 488 nm and emission at 525 nm was applied using a flow cytometer (Bio-Rad, United States).

### Immunofluorescence Assessment of NRF2 in H9C2 Cells

After treatment, H9C2 cells were fixed in 4% formaldehyde at room temperature for 20 min and 0.2% Triton X-100 for 5 min. After sealing with 5% BSA for 60 min, the cells were incubated with the anti-NRF2 (ab137550, Abcam, United Kingdom) at 4°C overnight. Subsequently, they were incubated with the secondary antibody (ZF-0315, ZSGB-BO, Beijing, China) in dark for 60 min. After staining with DAPI and 95% glycerin, images were obtained under an immunofluorescence microscope.

### Detection of Cell Transfection and Real-Time Quantitative PCR

H9C2 cells were seeded in 12-well plates for 24 h. The culture medium was replaced with serum-free medium (Opti-MEM I, Thermo Fisher Scientific, United States) after the cell density had reached 30%∼50%. Lipofectamine 2000 (Lipo2000, Thermo Fisher Scientific, United States) was diluted in serum-free medium and mixed with diluted siRNA (Gene Pharma, Shanghai, China) at room temperature for 20 min. The siRNA mixture was subsequently added to 12-well plates and incubated for 4∼6 h. The culture medium was immediately replaced with a complete medium, and the cells were continuously incubated for follow-up experiments. Total RNA was extracted from cells using an RNA isolation kit (R0027, Beyotime, Shanghai, China) and reverse transcribed into cDNA using a kit (FSQ-301, Toyobo life science, Japan). Quantitative real-time PCR (qPCR) was performed using the qPCR kit (Q411-02, Vazyme, Nanjing, China) according to the manufacture’s protocol. The 2−ΔΔCt method (Rao et al., 2013) was used to analyze the data and α-tubulin was used as an internal control.

The siRNA sequence of PKC was as follows: F:5′-GUAGUCACUGUACCGACUUTT-3′, R: 5′-AAG​UCG​GUA​CAG​UGA​CUG​CTT-3′. The primers were as follows: α-tubulin: F: 5′-CCCAACAAT GTGAAGACGG-3′, R: 5′-GCC​TCG​GTG​AAC​TCC​ATC​T-3′; PKC: F: 5′-GCGAAGCCCCTAAGA CAAT-3′, R:5′-CACCCCAGATGAAATCCCTAC-3′; NRF2: F: 5′-TGC​CCA​CAT​TCC​CAA​ACA AG-3′, R: 5′-TTG​CTC​CAT​GTC​CTG​CTG​TA-3′; HO-1: F: 5′-FTTCAGAAGGTCAGGTGTC-3′, R:5′-CTGTGTGGCTGGTGTGTAAG-3′.

### Measurement of PKC Overexpression

Phorbol 12-myristate 13-acetate (PMA, HY-18739, MCE, United States) was added to H9C2 cells and incubated for 24 h. Next, propofol or H_2_O_2_ was added to the culture as described above. Protein was extracted from the cells and analyzed by western blotting.

### Animal Using in the Experiments

The animal experiments were conducted in compliance with the principles for the Care and Use of Laboratory Animals of Shandong University, and the research protocol was approved by the Medical Ethics Committee for the Use of Experimental Animals at Shandong University (ECSBMSSDU2019-2-048). Wistar rats (weight 180∼240 g, 10 weeks old) were purchased from the Experimental Animal Center in Shandong University. The rats were housed at a constant room temperature (22∼24°C) with 45%∼55% humidity and fed with sterile water and a standard diet.

### Langendorff Perfused Heart Preparation

Rats were raised in the laboratory for three days before the experiments. After being anesthetized by intraperitoneal injection (IP) with 10% chloral hydrate, rat hearts were quickly dissected and immersed in ice-cold oxygenated KrebsHenseleit (KH) solution, which contained NaCl 115 mM, KCl 4.7 mM, MgSO_4_ 1.2 mM, CaCl_2_ 1.8 mM, KH_2_PO_4_ 1.2 mM, NaHCO_3_ 25 mM, and glucose 11 mM. Then, the hearts were quickly perfused in a retrofitted Langendorff system with KH solution at a constant velocity of 10 ml/min, which was gassed with 95% O_2_ and 5% CO_2_ at 37°C. The time between excision and suspension was limited to 1 min. After 15 min of stabilization, KH solution, propofol, or chelerythrine was perfused. Cardiac ischemia was induced by no-perfusion for 30 min and reperfusion was achieved by recanalization for 60 min. Coronary perfusion fluid was collected to measure LDH, SOD, and CK-MB. Hearts were kept in liquid nitrogen for 10 min before being stored at -80°C.

### Animal Grouping and Corresponding Treatment

After 15 min of stabilization, 24 hearts were randomly assigned to four groups: 1) C: hearts continuously pumped with KH solution for 120 min; 2) I/R: hearts continuously pumped with KH solution for 30 min and followed by cardiac ischemia for 30 min before reperfusion for 60 min; 3) I/R+P: hearts pumped with propofol (50 μM) for 20 min before ischemia: 4) I/R+P+CHE: hearts sequentially pumped with chelerythrine (10 μM) for 10 min and propofol for 20 min before ischemia (Figure 2B).

### Assessment of Lactate Dehydrogenase, Superoxide Dismutase, and Creatine Kinase-MB

The LDH and SOD levels of heart effluent were determined using assay kits (A020-2-2, A001-3-2, Nanjing Jian Cheng Bioengineering Institute, Nanjing, China). CK-MB was evaluated using an ELISA kit (SEKR-0059, Solarbio life sciences, Beijing, China). After reperfusion as described above, the effluent was plated in a 96-well plate, and the absorbance at 450 nm was determined on a Microplate Reader (Thermo Fisher Scientific, United States).

### Detection of Myocardium Infarct Size

After reperfusion as described above, hearts were frozen at -20°C for 30 min and cut into small sections (1∼2 mm), which were placed into 1% triphenyl tetrazolium chloride (TTC, T8877, Sigma, United States) at 37°C without light for 15 min. The sections were photographed using a camera and the images were quantified using picture analysis software (Image J, National Institutes of Health, United States). Myocardial infarction was measured by dividing the infarct area by the total area.

### Measurement of the Glutathione (GSH)/Oxidized Glutathione (GSSG) Ratio

The glutathione (GSH)/oxidized glutathione (GSSG) ratio of hearts was measured using a GSH/GSSG detection assay kit (S0053, Beyotime, Shanghai, China). Briefly, GSSG was restored to GSH by glutathione reductase, which was reacted with the chromogenic substrate DTNB to produce yellow TNB and GSSG. GSH was first eliminated by GSH scavenging auxiliary fluid, and then GSSG was measured using the above reaction principle. GSH was calculated by deducting GSSG from the total glutathione (GSSG+GSH). The reaction mixture was plated in a 96-well plate, and the absorbance at 410 nm was measured using a Microplate Reader (Thermo Fisher, United States).

### Histopathology Assessment

Hearts were immersed in 4% paraformaldehyde for 24 h and dehydrated conventionally for fixation in paraffin. The tissue was cut to a thickness of 3∼4 μm and dewaxed with xylene. The sections were then stained using a hematoxylin-eosin staining assay kit (HE, C0105S, Beyotime, Shanghai, China), and sealed with concentrated alcohol, xylene, and neutral resin. Images were acquired using a Nikon Eclipse 80i light microscope.

### Transmission Electron Microscopy Detection

The ultrastructure of myocardium mitochondria was examined by transmission electron microscopy (TEM, HITACHI, Japan). After reperfusion as described above, the left ventricle anterior wall was cut into 2 mm × 5 mm × 10 mm sections with sharp blades. The sections were immediately soaked in electron microscope fixative (G1102, Servicebio, Wuhan, China) for 3 h and incubated in 1% osmium 0.1 M phosphate buffer. Then, the sections were gradually dehydrated with ethanol and acetone, embedded in embedding solution, and baked in an oven. Finally, they were cut into 50∼80 nm and dyed with 2% uranyl acetate lead citrate. Images were acquired by TEM.

### Western Blot Analysis

RIPA lysis buffer (P0013B, Beyotime, Shanghai, China) with protease inhibitor (P0100, Solarbio life sciences, Beijing, China) was added to rat myocardial tissue or H9C2 cells to extract protein according to the manufacture’s protocol. The protein concentration was detected using a BCA Protein Assay Kit (P0012, Beyotime, Shanghai, China). Protein samples, which were mixed with loading buffer and heated at 100°C for 8 min, were separated by 12% SDS-PAGE gel electrophoresis and transferred to a polyvinylidene fluoride membrane (PVDF) pre-activated with methanol. The membrane was then blocked with 5% bovine serum albumin (BSA) for 1 h and immersed in the primary antibody solution overnight at 4°C. The primary antibodies were anti-NRF2, anti-HO-1 (E3F4S, CST, United States), anti-PKC (ab23511, Abcam, United Kingdom), anti-PKC (phosphor T497, ab59411, Abcam, United Kingdom), anti-α-tubulin (ab7291, Abcam, United Kingdom), and anti-H3 (17168-1-AP, Proteintech, Wuhan, China). The membranes were incubated with secondary antibodies (ZB-2301 or ZB-2305, ZSGB-BIO, Beijing, China) and the protein signals were detected using enhanced chemiluminescence (ECL) detection system. Images were analyzed using Image J software (National Institutes of Health, United States).

### Immunofluorescence and Immunohistochemistry Assessment in Myocardium

The heart paraffin sections dewaxing procedure was performed together with HE staining. Antigens were repaired with antigen retrieval solution (P0083, Beyotime, Shanghai, China) in the microwave for 20 min and blocked with 3% hydrogen peroxide (323381, Sigma, United States) for 10 min. After incubation with BSA for 60 min and the primary antibody (anti-NRF2) overnight, the sections were incubated with the secondary antibody (GTVision TM + polymer secondary antibody) for 30 min. Then the sections were stained with pre-prepared diaminobenzidine (DAB) staining solution (GK347011, Gene Tech, Shanghai, China) and hematoxylin for 3 min. Like HE staining, the sections were dehydrated and sealed. The images were photographed using a Nikon Eclipse 80i light microscope.

Using the same procedure described above, the sections were sealed with BSA and incubated with primary antibodies against anti-PKC or anti-PKC (phosphoT497) overnight. Subsequently, the sections were incubated with the secondary antibody (ZF-0315, ZSGB-BO, Beijing, China) for 60 min and sealed with DAPI or glycerin. Imagines were obtained under an immunofluorescence microscope.

### Statistical Analyses

All data are expressed as the mean ± standard (SD) deviation. The data analyses of perfusion liquid were handled by two-way ANOVA with Bonferroni’s correction and other data were analyzed by one-way ANOVA followed by Tukey’s post hoc test or T-test. GraphPad Prism 8.0 (GraphPad Software, Chicago, United States) was used for statistical analysis, and *p* < 0.05 served as an index of statistical significance.

## Results

### Effect of Propofol on H_2_O_2_-Induced H9C2 Cell Injury

**FIGURE 3:**
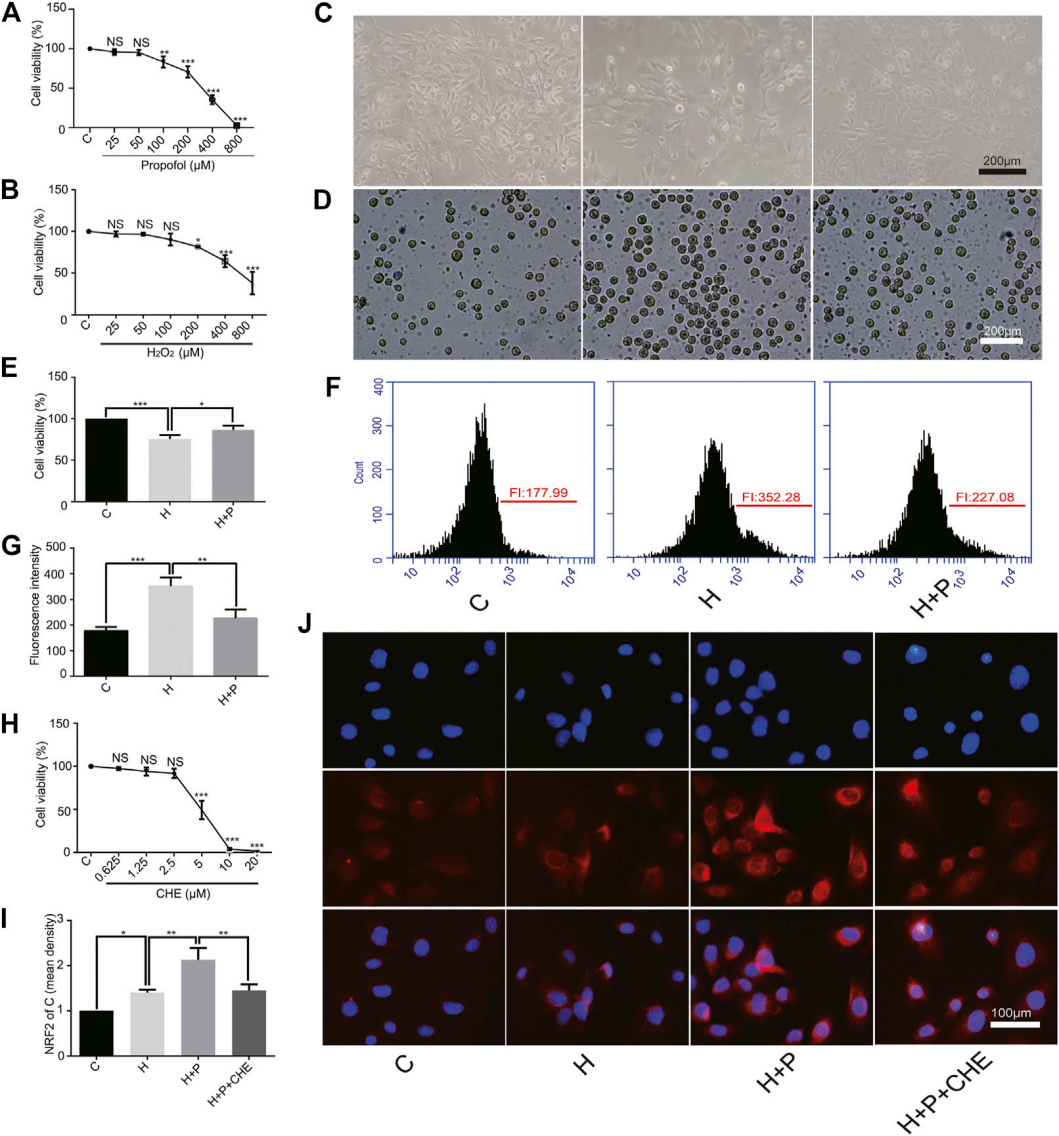
Effect of propofol on H_2_O_2_-induced H9C2 cells injury. Cell viability of H9C2 cells treated with H_2_O_2_ (H, 0∼800 μM) **(A)** and propofol (P, 0∼800 μM) **(B)**. **(C–E)**: Effect of propofol on H_2_O_2_-induced cell deaths. Dead cells were stained with blue by trypan blue. Scale bar: 200 μm. **(F)** and **(G)**: Effect of propofol on H_2_O_2_-induced ROS production. Quantification of ROS is expressed as the fluorescence intensity (FI). **(H)**. Viability of H9C2 cells treated with chelerythrine (CHE, 0∼20 µM). **(I,J)**. Expression of NRF2 in the H9C2 cells based on the immunofluorescence assay. The scale bar is 100 µM. N = 3. Data are expressed as the mean ± SD. Significance was calculated using the ANOVA and *p* < 0.05 was considered statistically significant. *p < 0.05, **p < 0.01, ***p < 0.001.

The CCK-8 assay showed that propofol (35.53 ± 5.69) or H_2_O_2_ (64.30 ± 7.19) caused severe cell damage at 400 μM (Figures 3A,B). The present study was to explore the underlying mechanism of propofol on myocardial protection by antioxidation. Therefore, 50 μM (95.30 ± 3.42) propofol and 200 μM (81.70 ± 0.90) H_2_O_2_ were selected for the follow-up experiments. A phase-contrast microscope and the trypan blue test showed that propofol increased H9C2 cell viability in the H+P group (H+P, 86.70 ± 4.850) compared with the H group (H, 75.53 ± 4.87) (Figures 3C–E). Flow cytometry also showed that propofol abated ROS production in the H+P group (H+P, 227.1 ± 33.79) compared with the H group (H, 352.3 ± 32.98) (Figures 3F,G).

### Change of NRF2 Nuclear Translocation in H9C2 Cells by Propofol

CCK-8 assay showed that chelerythrine caused severe damage to H9C2 cells at concentrations ranging from 10∼20 μM (10, 4.03 ± 1.36.20, 1.53 ± 0.15). Therefore, 5 μM (5, 49.27 ± 10.72) was used in the follow-up experiments (Figure 3H). H9C2 cell immunofluorescence showed that H_2_O_2_ promoted NRF2 nuclear translocation in the H group (H, 1.40 ± 0.07) compared with the C group (C, 1). Propofol (H+P, 2.13 ± 0.26) preconditioning significantly increased NRF2 nuclear translocation compared with the H group. Propofol plus chelerythrine (H+P+CHE, 1.45 ± 0.13) preconditioning reduced NRF2 nuclear translocation compared with propofol alone (Figures 3I,J).

### Effect of Propofol on PKC Knockdown in H9C2 Cells

**FIGURE 4:**
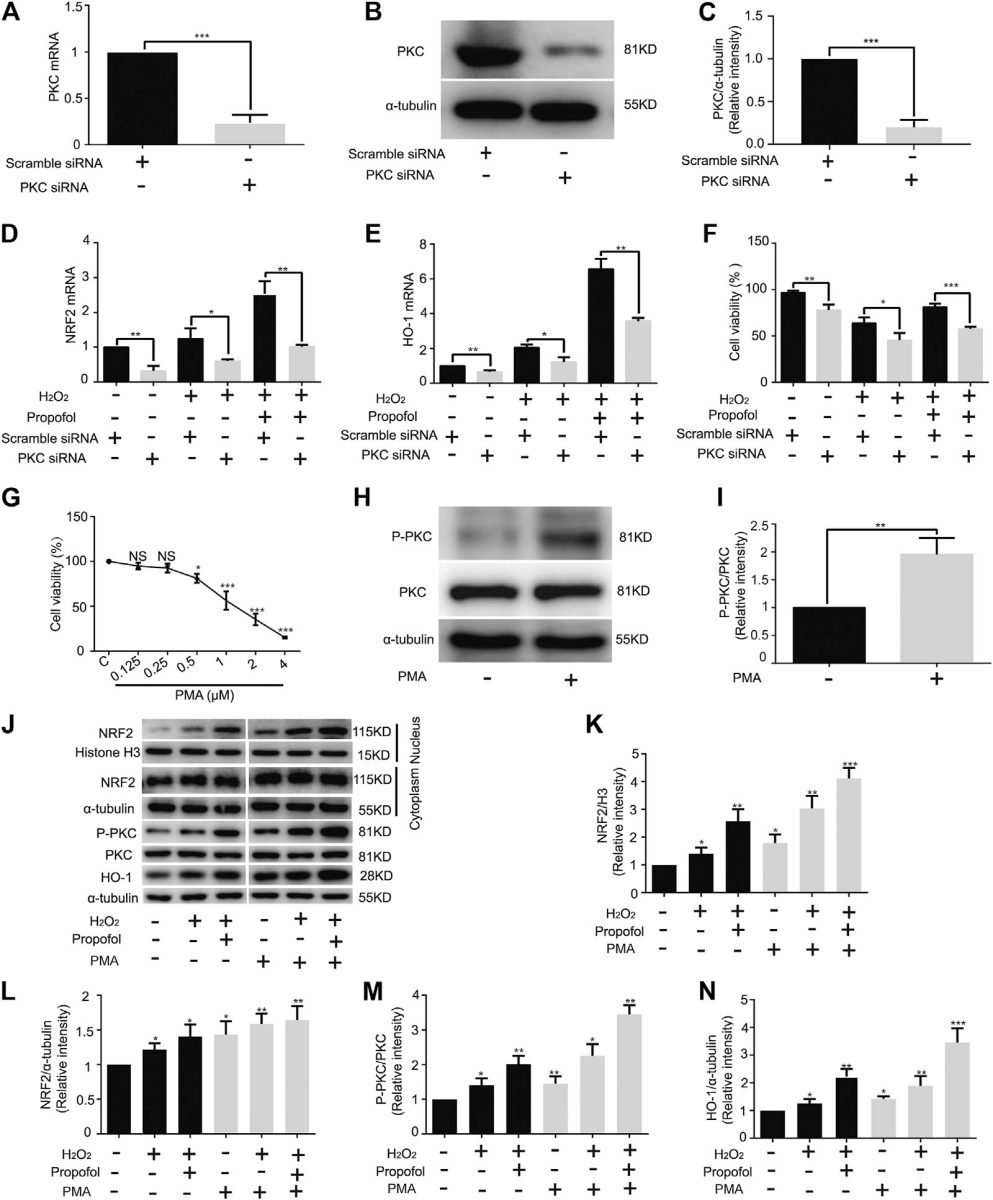
Influence of propofol on PKC knockdown and overexpression in H9C2 cells. Expression of PKC mRNA **(A)** and protease **(B,C)** in H9C2 cells transfected with PKC and scramble siRNA. Levels of NRF2 **(D)** and HO-1 **(E)** mRNAs in H9C2 cells transfected with a scramble and PKC siRNA. **(F)**. Influence of propofol on H9C2 cell viability of PKC knockdown under oxidative stress condition. **(G)**. Viability of H9C2 cells treated with phorbol 12-myristate 13-acetate (PMA, 0∼4 µM). **(H,I)**. Expression of PKC in H9C2 cells treated with 0.5 µM PMA based on western blot assay. **(J–N)**. Influence of propofol on H9C2 cell PKC, NRF2 and HO-1 expressions of PKC overexpression under oxidative stress condition. Data are expressed as the mean ± SD. Significance was calculated using the ANOVA or Student’s t-test. *p* < 0.05 was considered statistically significant. *p < 0.05, **p < 0.01, ***p < 0.001.

Knockdown of PKC significantly inhibited PKC mRNA expression (Scramble, 1. PKC, 0.24 ± 0.09) (Figure 4A). Western blotting showed that siRNA effectively knock down PKC protease, just as mRNA (Scramble, 1. PKC, 0.20 ± 0.08) (Figures 4B,C). The mRNA levels of NRF2 (C: Scramble, 1; PKC, 0.32 ± 0.14. H: Scramble, 1.24 ± 0.30; PKC, 0.6 ± 0.05. H+P: Scramble, 2.53 ± 0.37; PKC, 1.01 ± 0.06) and HO-1 (C: Scramble, 1; PKC, 0.65 ± 0.10. H: Scramble, 2.06 ± 0.18; PKC, 1.22 ± 0.27. H+P: Scramble, 6.56 ± 0.59; PKC 3.58 ± 0.18) were significantly reduced by PKC siRNA (Figures 4D,E). The CCK8 assay demonstrated that propofol inhibited H_2_O_2_-induced cell damage, but did not protect PKC siRNA transfected H9C2 cells from injury (Scramble: C, 96.96 ± 1.99; H, 63.94 ± 6.01; H+P, 81.57 ± 3.38. PKC: C 77.98 ± 5.78; H, 45.4 ± 7.91; H+P, 57.64 ± 2.27) (Figure 4F).

### Influence of Propofol on PKC Overexpression in H9C2 Cells

The CCK-8 assay showed that 1 µM PMA (56.37 ± 10.22) caused H9C2 cell damage (Figure 4G). Therefore, 0.5 µM PMA was used in the follow-up experiments. The western blot assay showed that PMA significantly improved PKC phosphorylation in H9C2 cells (C, 1; PMA, 1.96 ± 0.29) (Figures 4H,I). With H_2_O_2_-induced oxidation, propofol increased NRF2 nuclear translocation (C, 1. H, 1.41 ± 0.22. H+P, 2.57 ± 0.44. PMA: C, 1.79 ± 0.30. H, 3.04 ± 0.45. H+P, 4.12 ± 0.39) (Figures 4J,K) and HO-1 expression (C, 1. H, 1.40 ± 0.20. H+P, 2.01 ± 0.24. PMA: C, 1.44 ± 0.22. H, 2.25 ± 0.34. H+P, 3.45 ± 0.27) (Figures 4J,N) in H9C2 cells. The changes of PKC phosphorylation are similar to that of HO-1 expression (Figures 4J,M).

### Influence of Propofol on Levels of LDH, SOD and CK-MB in Coronary Effluent

**FIGURE 5:**
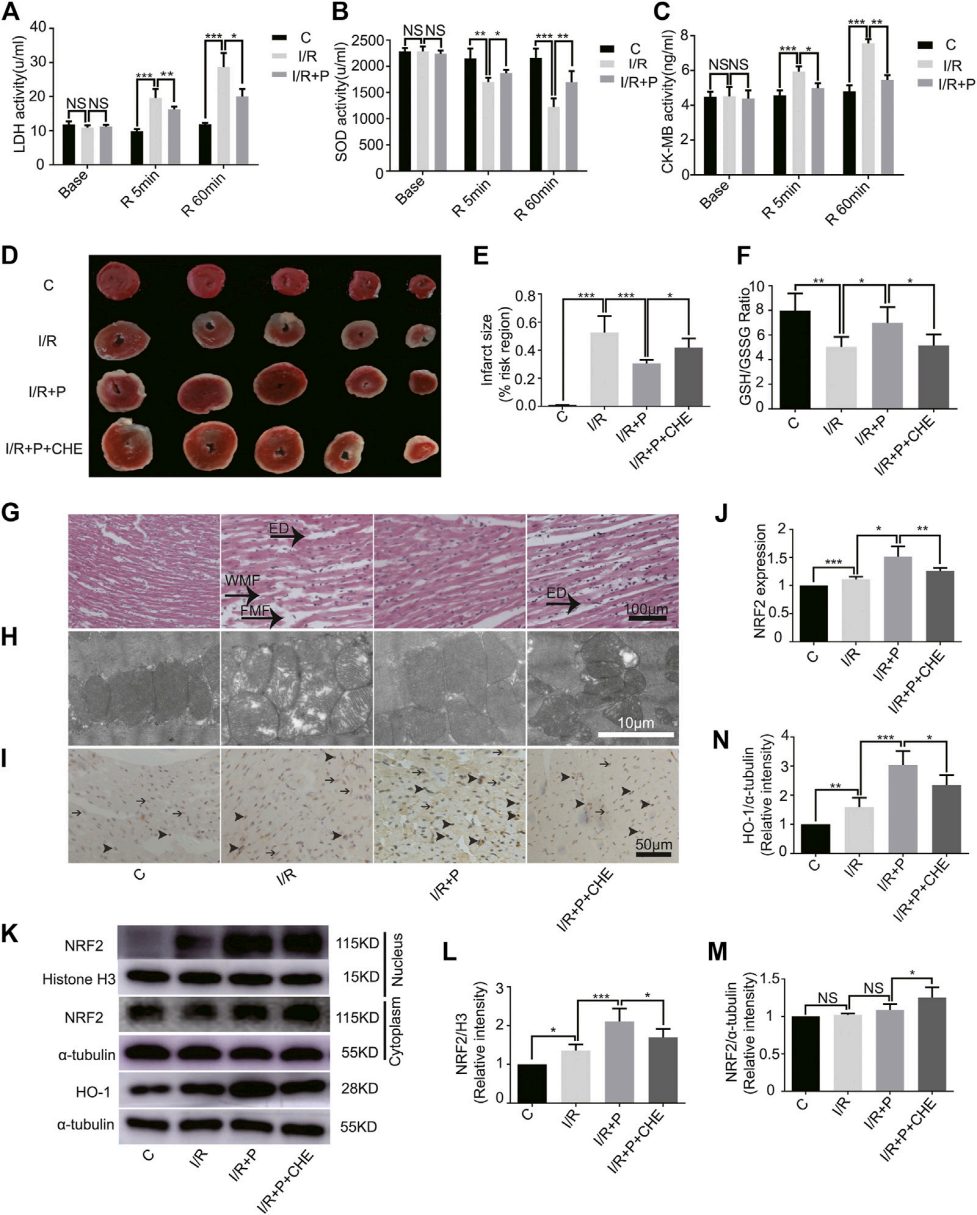
Effect of propofol on I/R-induced myocardium injury. Expressions of LDH (**A)**, SOD (**B)**, and CK-MB (**C)** in the coronary perfusate. The perfusate was collected before ischemia (base) and reperfusion for 5 min (R 5 min) and 60 min (R 60 min). **(D,E)**: Myocardial infarction areas were measured by triphenyltetrazolium chloride (TTC) staining. **(F)**. GSH/GSSG changes in myocardium. **(G)**. Structural changes in myocardial fibers based on HE staining. ED (edema), WMF (wavy myofibers), and FMF (faulted myofibers). The scale bar is 100 µm. **(H)**. Mitochondrial changes under electron microscopy. The scale bar is 10 µm. **(I,J)**. The expression of NRF2 in myocardium based on immunohistochemistry assay. The arrowhead represents nuclear expression of NRF2, and the arrow represents cytoplasmic expression. The scale bar is 100 µm. **(K–N)**. Expressions of NRF2 and HO-1 in myocardium based on the western blot assay. N = 6. The data are expressed as the mean ± SD. Significance was calculated using two-way ANOVA with Bonferroni’s correction. *p* < 0.05 was considered statistically significant. *p < 0.05, **p < 0.01, ***p < 0.001.

At baseline, no significant differences in the levels of LDH (C, 11.82 ± 0.92; I/R, 10.92 ± 0.65; I/R+P, 11.18 ± 0.56), CK-MB (C, 4.49 ± 0.30; I/R, 4.52 ± 0.53; IR+P, 4.39 ± 0.47) and SOD (C, 2282 ± 67.39; I/R, 2282 ± 96.58; I/R+P, 2240 ± 59.29) were detected. In group I/R, however, levels of LDH (I/R, 19.63 ± 2.59) or CK-MB (I/R, 5.94 ± 0.31) were significantly higher and SOD (I/R, 1699 ± 83.04) was significantly lower after 5 min of reperfusion compared with group C (LDH: C, 9.89 ± 0.60. CK-MB: C, 4.57 ± 0.30. SOD: C, 2151 ± 187.6). The changes in these indexes were more obvious after reperfusion 60 min (LDH: I/R, 28.73 ± 4.10. CK-MB: I/R, 5.94 ± 0.31. SOD: I/R, 1226 ± 159.7). All of these changes were neutralized by propofol (I/R+P. LDH: R 5 min, 16.27 ± 0.81; R 60 min, 20.06 ± 2.167. CK-MB: R 5 min, 4.98 ± 0.29; R 60 min, 5.46 ± 0.28. SOD: R 5 min, 1870 ± 64.21; R 60 min, 1697 ± 212.8) (Figures 5A–C).

### I/R Myocardium Injury by Activating PKC With Propofol Pre-Treatment

The infarcted myocardial tissues showed pale staining, while un-infarcted tissues were red. Compared with group C (C, 0.01 ± 0.00), significant expansions of infarcted areas were observed in group I/R (I/R, 0.53 ± 0.12). Propofol treatment reduced the infarcted areas (I/R+P, 0.31 ± 0.02), but chelerythrine (I/R+P+CHE, 0.42 ± 0.07) increased those areas compared with group I/R+P (Figures 5D,E).

The GSH/GSSG ratio of myocardial tissues was significantly lower in group I/R (I/R, 5.04 ± 0.82) than group C (C, 7.99 ± 1.39). Propofol (I/R+P, 6.98 ± 1.29) decreased the I/R tissues GSH/GSSG ratio, an effect that was reduced by chelerythrine (I/R+P+CHE, 5.46 ± 0.84) (Figure 5F).

HE staining revealed obvious edema (ED), wavy myofibers (WMF), and faulted myofibers (FMF) in the left ventricular area of group I/R compared with group C. The myocardial tissue injury was significantly reduced in group I/R+P compared with group I/R. However, the injury was significantly increased in group I/R+P+CHE compared with group I/R+P (Figure 5G). TEM showed that distorted or enlarged myocardial mitochondria and mitochondrial ridges in group I/R compared with group C. These mitochondrial injuries were obviously alleviated in group I/R+P compared with group I/R. Chelerythrine attenuated these effects (Figure 5H).

### Change of Nuclear Translocation of NRF2 and HO-1 Expression by Propofol

Immunohistochemistry revealed increased nuclear expression of NRF2 in group I/R (I/R, 1.11 ± 0.05) compared with group C (C, 1). Propofol also promoted NRF2 expression in group I/R+P (I/R+P, 1.51 ± 0.18) compared with group I/R. Additionally, NRF2 levels were significantly in group I/R+P+CHE (I/R+P+CHE, 1.26 ± 0.06) than in group I/R+P (Figures 5I,J).

The protein level of NRF2 in the myocardial nucleus, which was much lower in group C (C, 1) than in all other groups, was significantly higher than in group I/R (I/R, 1.36 ± 0.15). Additionally, the level was significantly lower in group I/R+P+CHE (I/R+P+CHE, 1.70 ± 0.22) than group I/R+P (I/R+P, 2.10 ± 0.34) (Figures 5K,L). There were no differences in protein levels in the myocardial cytoplasm across the groups (C, 1. I/R, 1.02 ± 0.02. I/R+P, 1.09 ± 0.08. I/R+P+CHE, 1.25 ± 0.14) (Figures 5K,M). Protein expression of HO-1 (C, 1. I/R, 1.59 ± 0.33. I/R+P, 3.04 ± 0.48. I/R+P+CHE, 2.35 ± 0.34) in myocardial tissue was similar to NRF translocation in the nucleus (Figures 4K,N).

### Change of PKC Activity by Propofol

**FIGURE 6:**
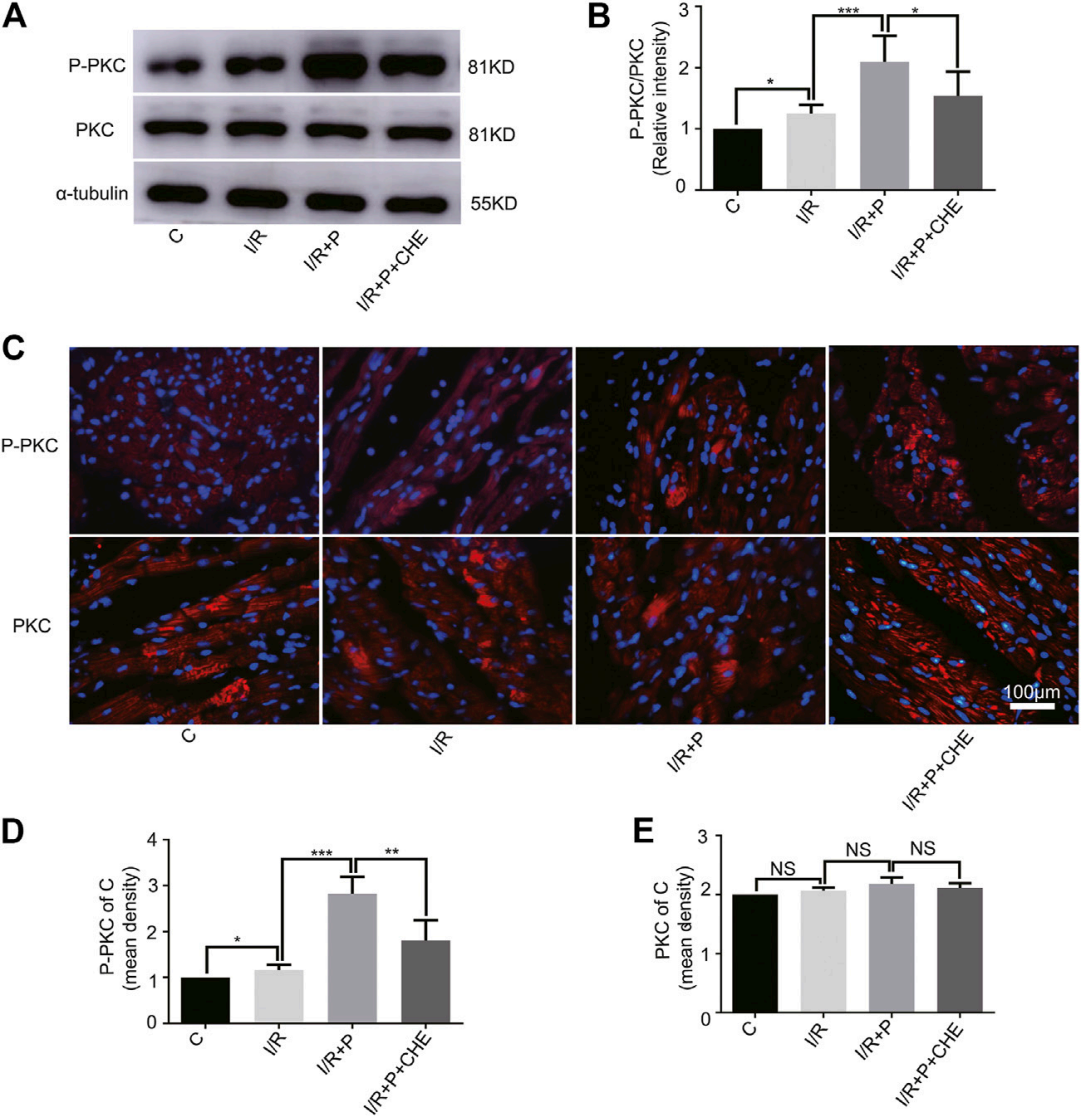
Change of PKC activity by propofol. **(A,B)**. Expressions of PKC and P-PKC in myocardium based on the western blot assay. **(C–E)**. Expressions of PKC and P-PKC in myocardium based on immunofluorescence. N = 6. Data are expressed as the mean±SD. The scale bar is 100um. Significance was calculated using the ANOVA. *p* < 0.05 was considered statistically significant. *p < 0.05, **p < 0.01, ***p < 0.001.

The western blot assay showed that PKC phosphorylation was slightly activated by I/R (C, 1. I/R, 1.25 ± 0.14) and significantly activated by propofol (I/R+P, 2.10 ± 0.43). This effect was much lower in group I/R+P+CHE (I/R+P+CHE, 1.54 ± 0.39) than group I/R+P (Figures 6A,B). These results were consistent with the NRF2 nuclear translocation. The immunofluorescence results were consistent with the western blot results (P-PKC/C. C, 1. I/R, 1.16 ± 0.11. I/R+P, 2.82 ± 0.38. I/R+P+CHE, 1.81 ± 0.44. PKC/C. C, 1. IR, 1.04 ± 0.02. I/R+P, 1.10 ± 0.06. I/R+P+CHE, 1.06 ± 0.04) (Figures 6C–E).

## Discussion

This study demonstrated that propofol preconditioning protected H9C2 cells from H_2_O_2_-induced injury and myocardium from I/R injury in Langendorff hearts in rats, which is consistent with previous studies (Lai et al., 2011; Ha et al., 2012; King et al., 2012). In some clinical operations, such as heart transplantation (Yang et al., 2015), aortic surgery (Kumagai et al., 2006), or coronary artery bypass grafting surgery (Pan et al., 2019), myocardial IR injury is a common cause of damage to patients’ hearts. Previous studies have shown that propofol exerts a cardio-protective effect on myocardial IR injury through the MAPK/ERK pathway, repressing the JAK/STAT signaling pathway and suppressing the TRPV4 channel with subsequent inhibition of intracellular Ca^2+^ overload (Halladin, 2015; Wang et al., 2019; Yan and Qi, 2019). However, the underlying mechanism has not been fully elucidated. Therefore, preventing I/R injury is critically important. Peter J’s (Wickley et al., 2006) research groups demonstrate that propofol activates PKC in rat ventricular myocytes. MIAN GE’ (Ge et al., 2015) research groups confirm that propofol activates NRF2 in the kidney in rat orthotopic liver autotransplantation (OLAT) models. In contrast to these studies, herein we focused on whether propofol preconditioning could increase nuclear translocation of NRF2 and HO-1 expression by activating PKC, which is the major contribution of the work.

The results suggested that the PKC/NRF2 anti-oxidative stress signaling pathway was involved in myocardial protection. Under oxidative stress, NRF2 is activated by PKC and translocated from the cytoplasm into the nucleus (Espada and Ana, 2009). Then NRF2 binds to antioxidant reaction elements (AREs) to promote the production of antioxidant enzymes such as heme HO-1, superoxide dismutase (SOD), or catalase (Zhang et al., 2015). H_2_O_2_ or I/R can destroy antioxidant enzymes, leading to the accumulation of reactive oxygen species (ROS) and cell injury (Matés, 2000). HO-1 has antioxidant function through catalyzing hemoglobin degradation (Yet et al., 2001). The present research suggested that propofol improved the antioxidant capacity of myocardial cells by increasing HO-1 expression and reducing LDH or CK-MB expression.

An *in vitro* study showed that propofol could effectively inhibit apoptosis and protect cardiomyocytes from fatal injury (Kim et al., 2008). Oxidative stress has been shown to induce apoptosis (Tsutsui et al., 2011), and NRF2 inhibits cell apoptosis by increasing the expression of anti-apoptotic factors such as Bcl-2 or reducing the expression of apoptotic factors such as caspase-3 (Niture and Jaiswal, 2012). Similarly, previous studies have shown that downregulation of NRF2 aggravates myocardial cell dysfunction (Erkens et al., 2015), and upregulation of NRF2 prevents H9C2 damage induced by H_2_O_2_ (Shinjo et al., 2018). In present study showed that myocardial I/R injury slightly increased NRF2 nuclear translocation, which was significantly promoted by propofol.

PKC plays an important role in NRF2 nuclear translocation (Smith et al., 2016). The association between PKC and NRF2 activation has been reported (Díaz-Ruíz et al., 2019). In a zebrafish model, Wang et al. report that PKC is involved in NRF2 activation (Wang et al., 2020). In the study, propofol preconditioning plus PKC inhibitor still exhibited myocardial protection compared with the C group, suggesting that other molecules or pathways in addition to PKC were involved in myocardial protection. It has been reported that propofol protects cardiomyocytes from hypoxia/reoxygenation-induced deaths by up-regulating the expression of heme HO-1 (Kobayashi et al., 2008). Other studies have demonstrated clearance of free radicals and inhibition of mitochondrial membrane permeability changes (Javadov et al., 2000). Protection against calcium overload (Nakae et al., 2000), inhibition of IL-8 release from neutrophils (Galley et al., 1998), and overproduction of NO in endothelial cells (Park et al., 1995) are involved in myocardial protection of propofol in I/R injury. These results were consistent with previous studies, in which mitogen-activated protein kinase (MAPK) and phosphatidyl-inositol-3 kinase modulate NRF2 activity (Dang et al., 2019). Additionally, as a nonspecific inhibitor of PKC, chelerythrine may not block all PKC subtypes (Keenan et al., 1997).

The research had some limitations. First, the study only conducted western blotting and immunofluorescence of PKC but did not specify PKC, because there are many subtypes of PKC and the relationship between them is complex (Wickley et al., 2006). Second, pre-protection effect changes induced by propofol on the myocardium in a time-dependent manner were not explored. The pre-protection time of propofol was set to 20 min according to a previous study (Ebel et al., 1999), which has shown that 20 min of propofol pre-treatment is suitable to protect myocardial against I/R-induced injury in rats Langendorff heart model. We hope the molecular mechanism underlying the myocardial protection of propofol will be further studied in the future.

## Data Availability

The original contributions presented in the study are included in the article/Supplementary Material, further inquiries can be directed to the corresponding authors.
