# Supplementary figures and images for: Propofol Inhibits Ischemia/Reperfusion-Induced Cardiotoxicity Through the Protein Kinase C/Nuclear Factor Erythroid 2-Related Factor Pathway
Source: Front Pharmacol. 2021 May 13;12:655726. doi: 10.3389/fphar.2021.655726 (PMC8155638; doi:10.3389/fphar.2021.655726)

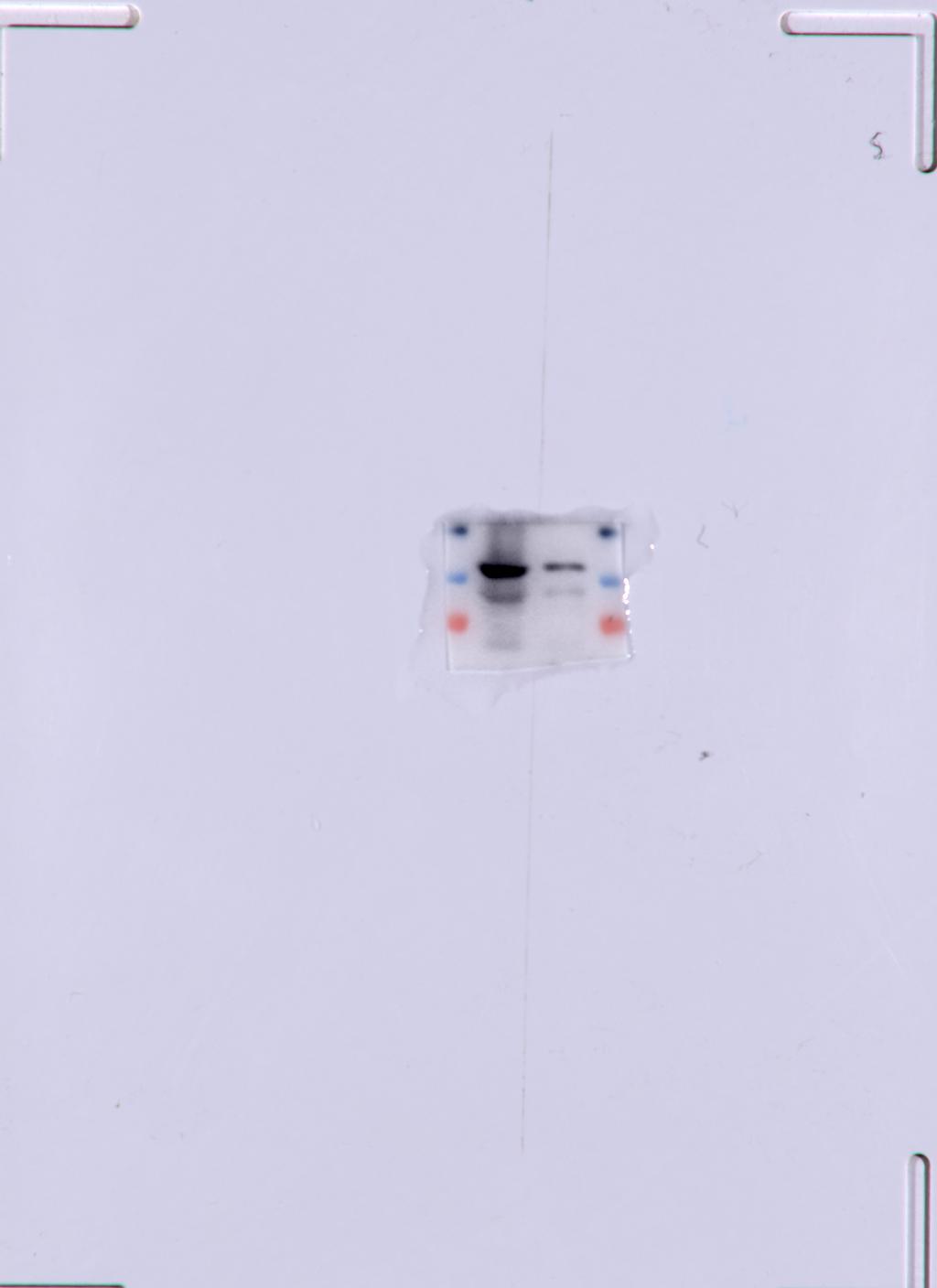

Supplement: Supplementary file 1 [file DataSheet1.ZIP › Supplementary_Material/Fig 4 B, H, J/Fig 4 B/Fig 4 B PKC.jpg]

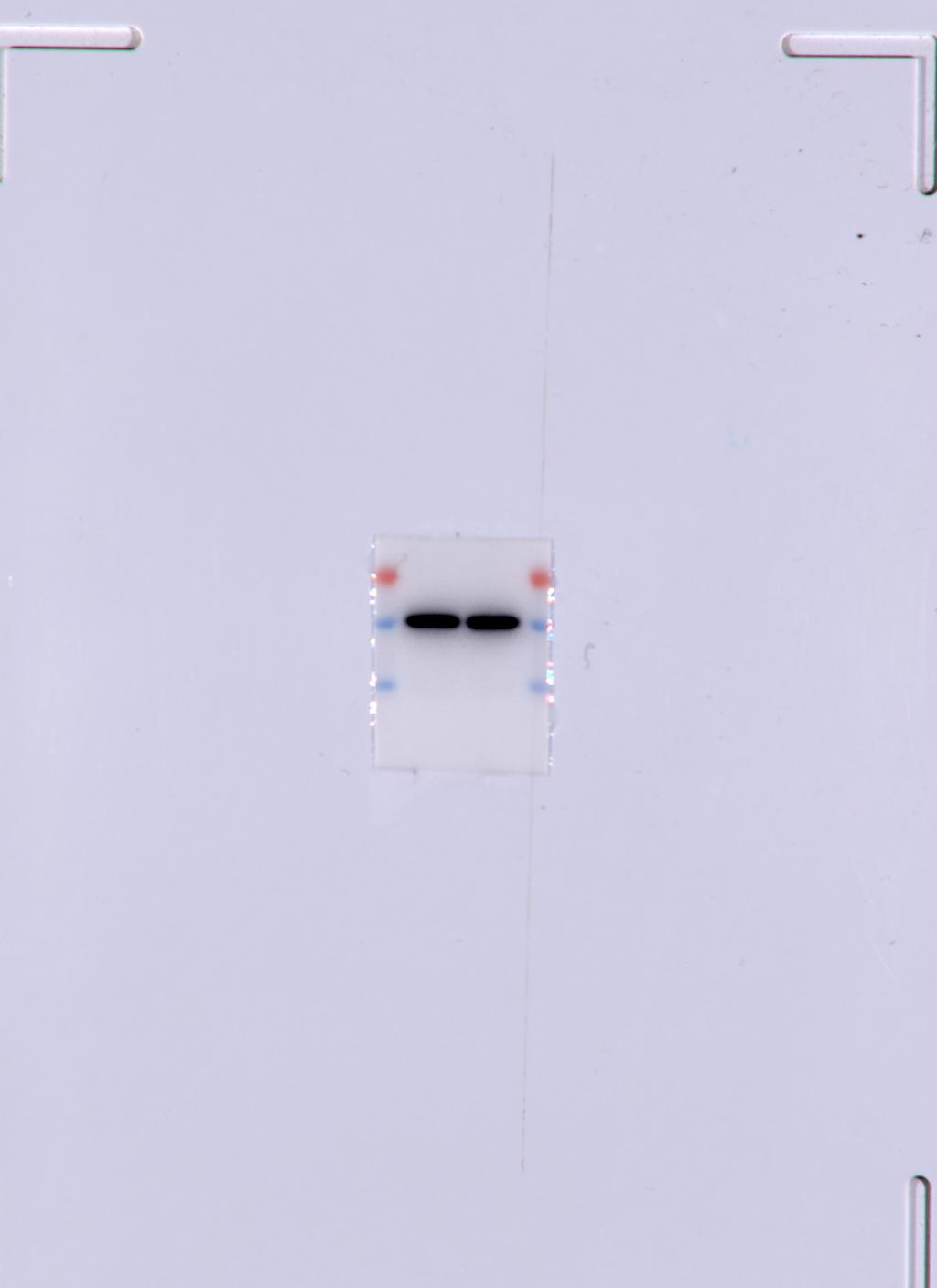

Supplement: Supplementary file 1 [file DataSheet1.ZIP › Supplementary_Material/Fig 4 B, H, J/Fig 4 B/Fig 4 B α-tubulin.jpg]

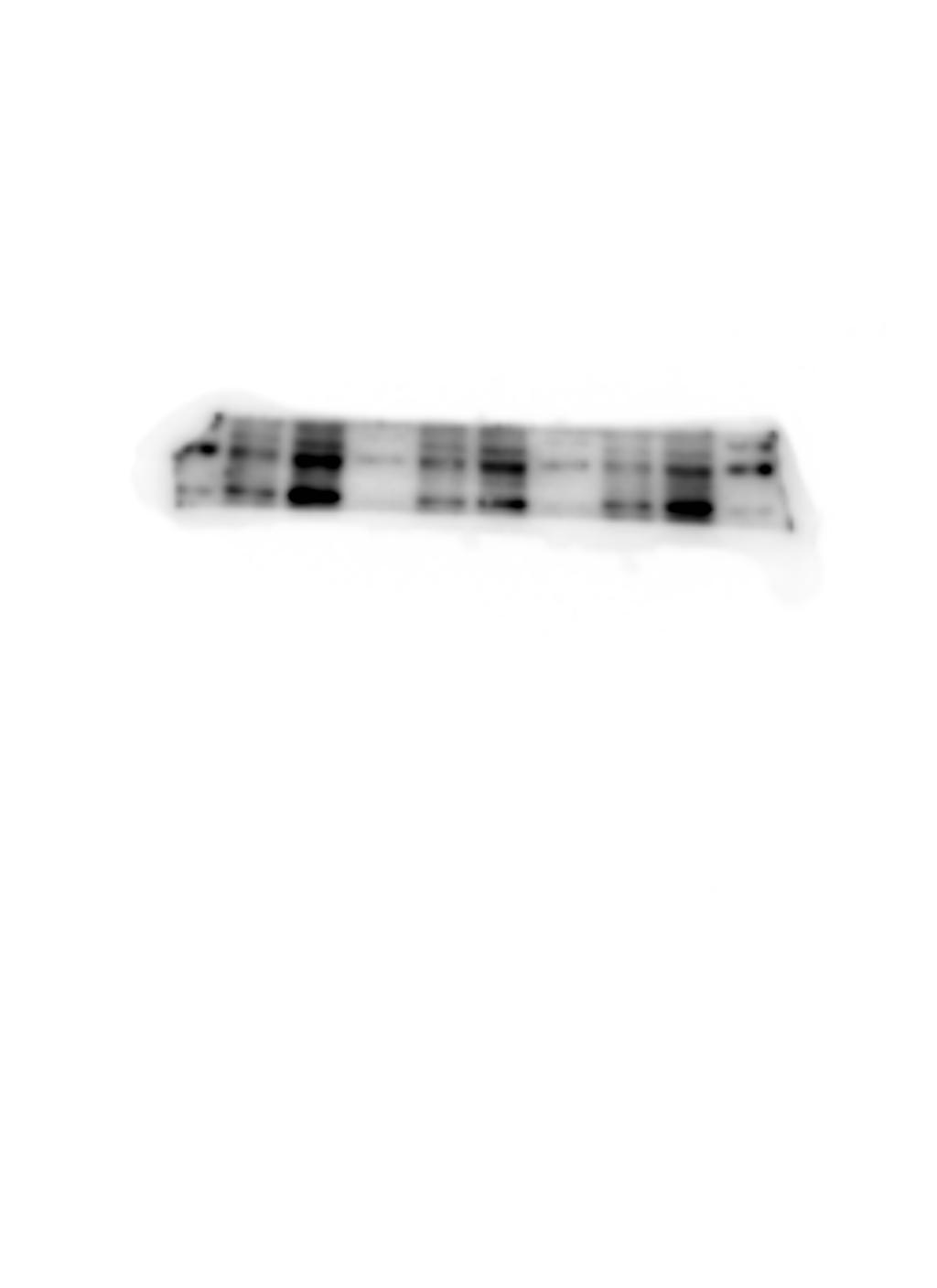

Supplement: Supplementary file 1 [file DataSheet1.ZIP › Supplementary_Material/Fig 4 B, H, J/Fig 4 H/Fig 4 H p-pkc.jpg]

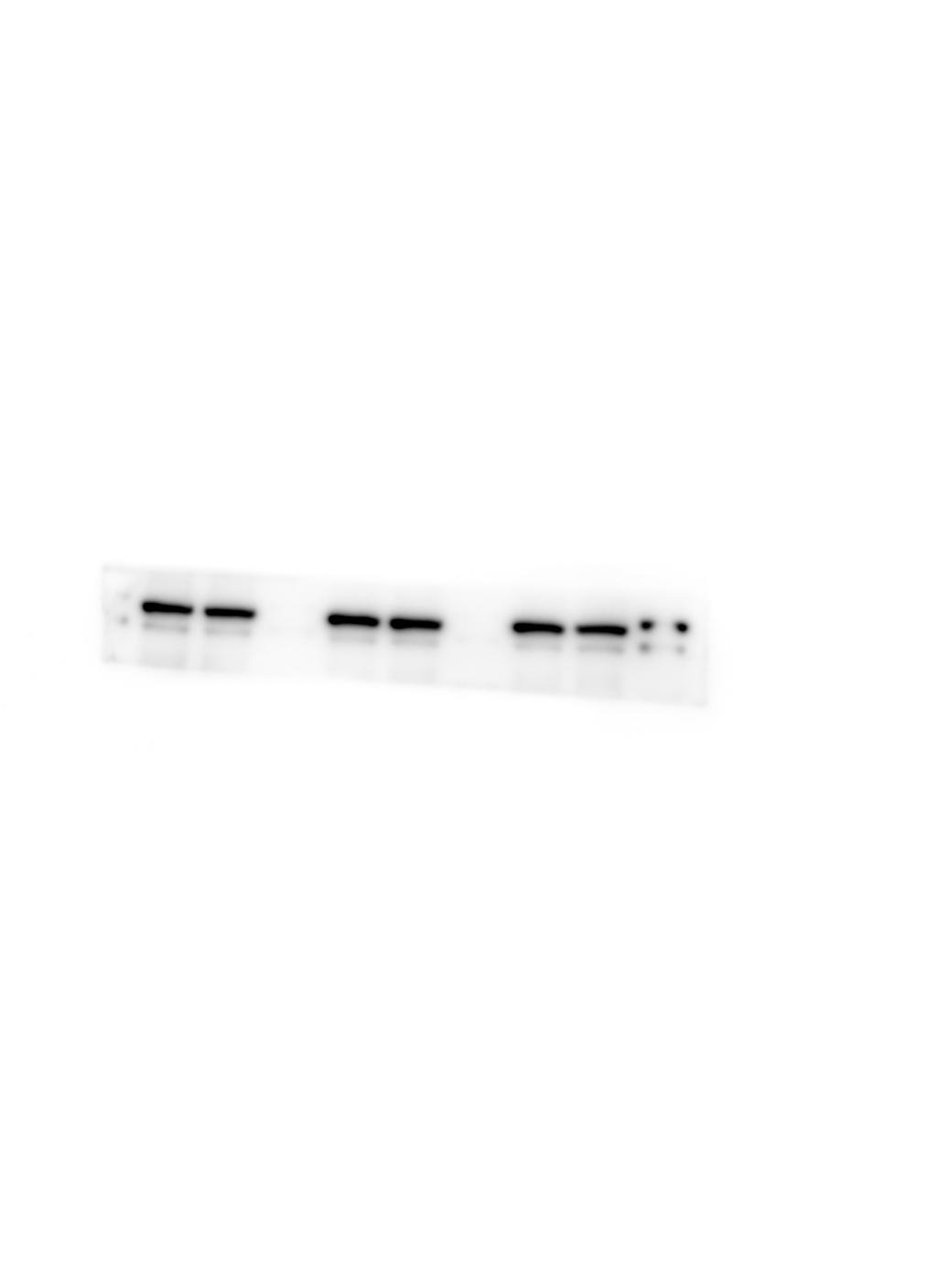

Supplement: Supplementary file 1 [file DataSheet1.ZIP › Supplementary_Material/Fig 4 B, H, J/Fig 4 H/Fig 4 H pkc.jpg]

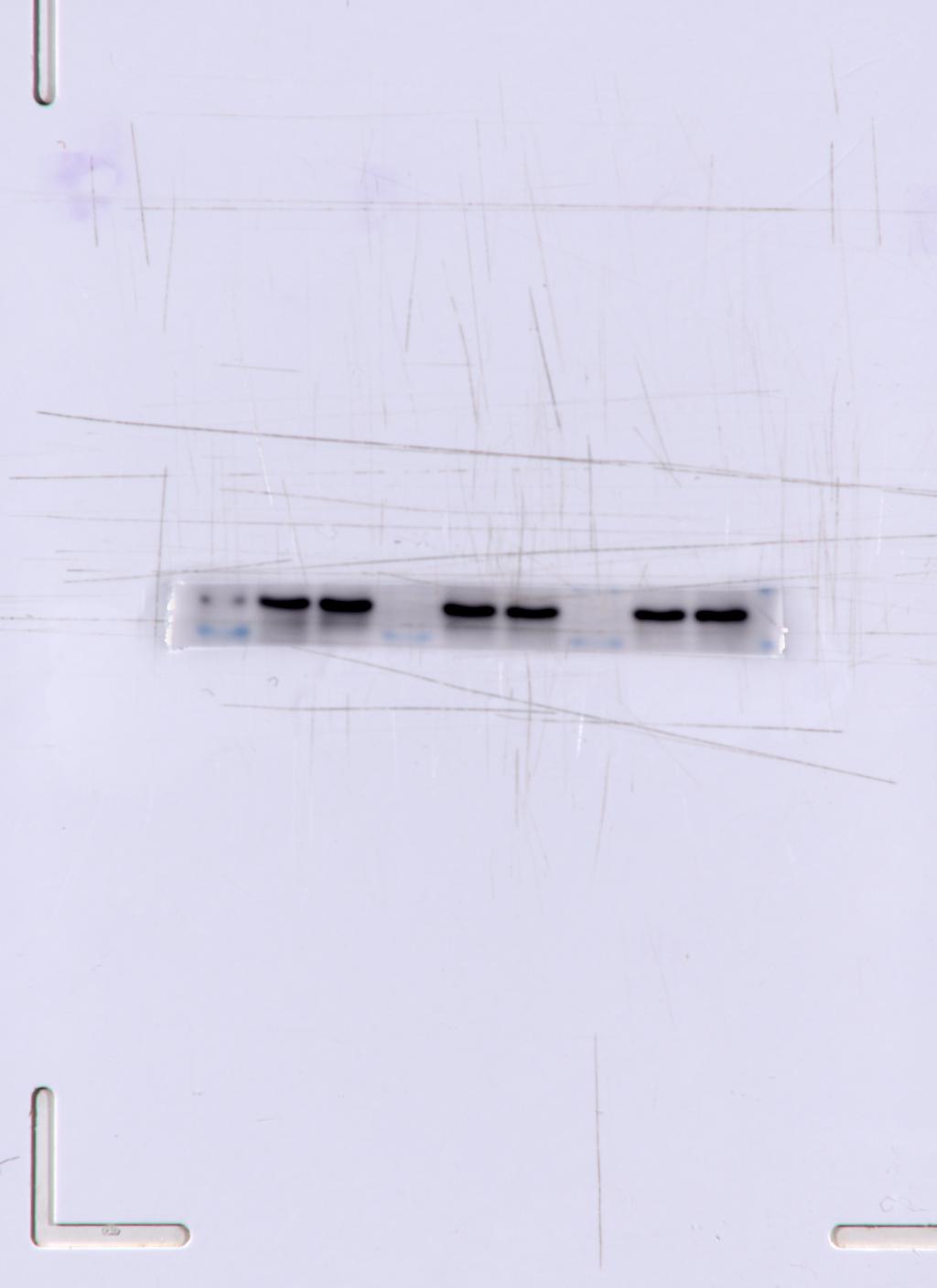

Supplement: Supplementary file 1 [file DataSheet1.ZIP › Supplementary_Material/Fig 4 B, H, J/Fig 4 H/Fig 4 H α-tubulin.jpg]

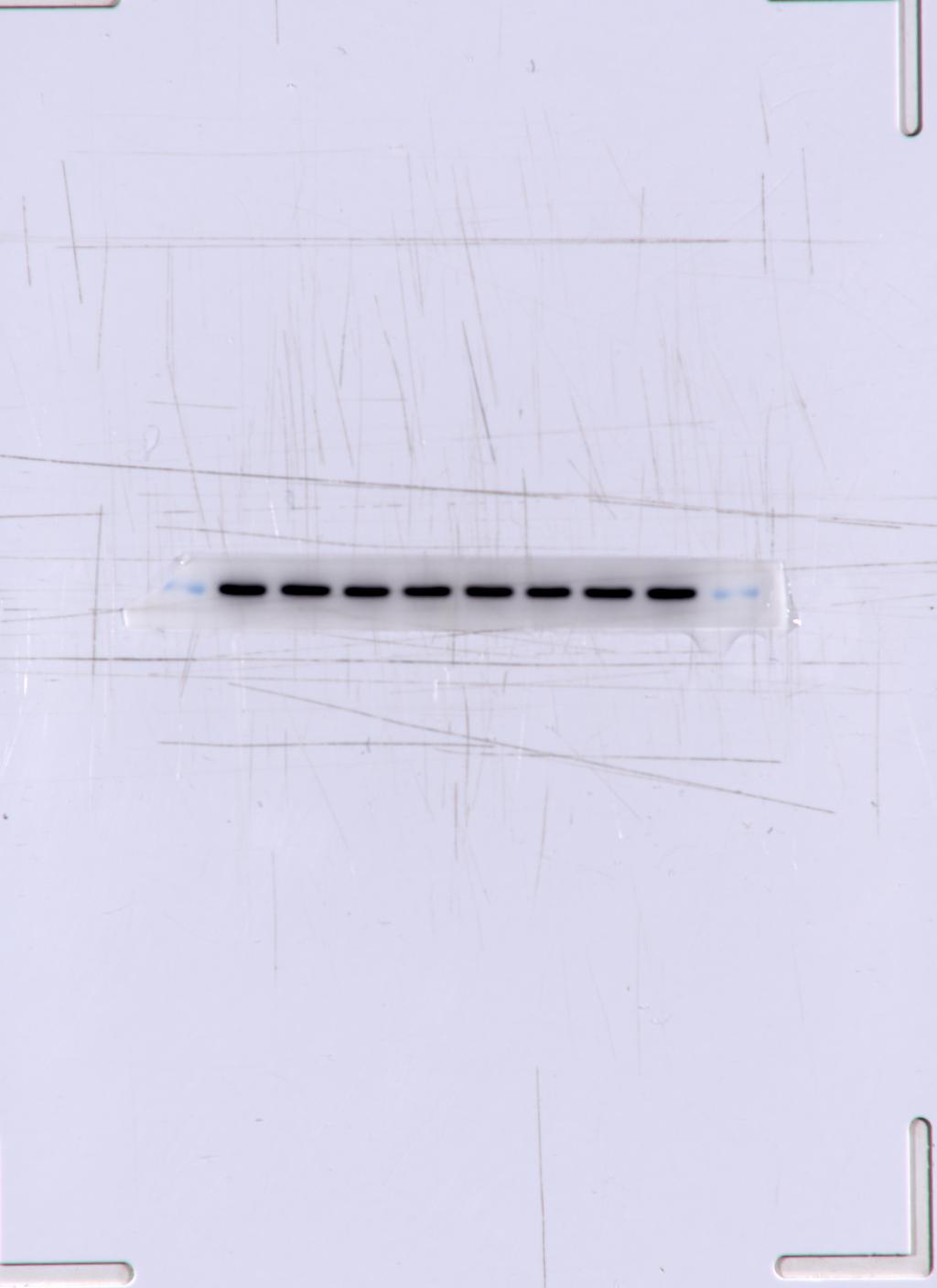

Supplement: Supplementary file 1 [file DataSheet1.ZIP › Supplementary_Material/Fig 4 B, H, J/Fig 4 J/Fig 4 J Histone H3 Nuleus.jpg]

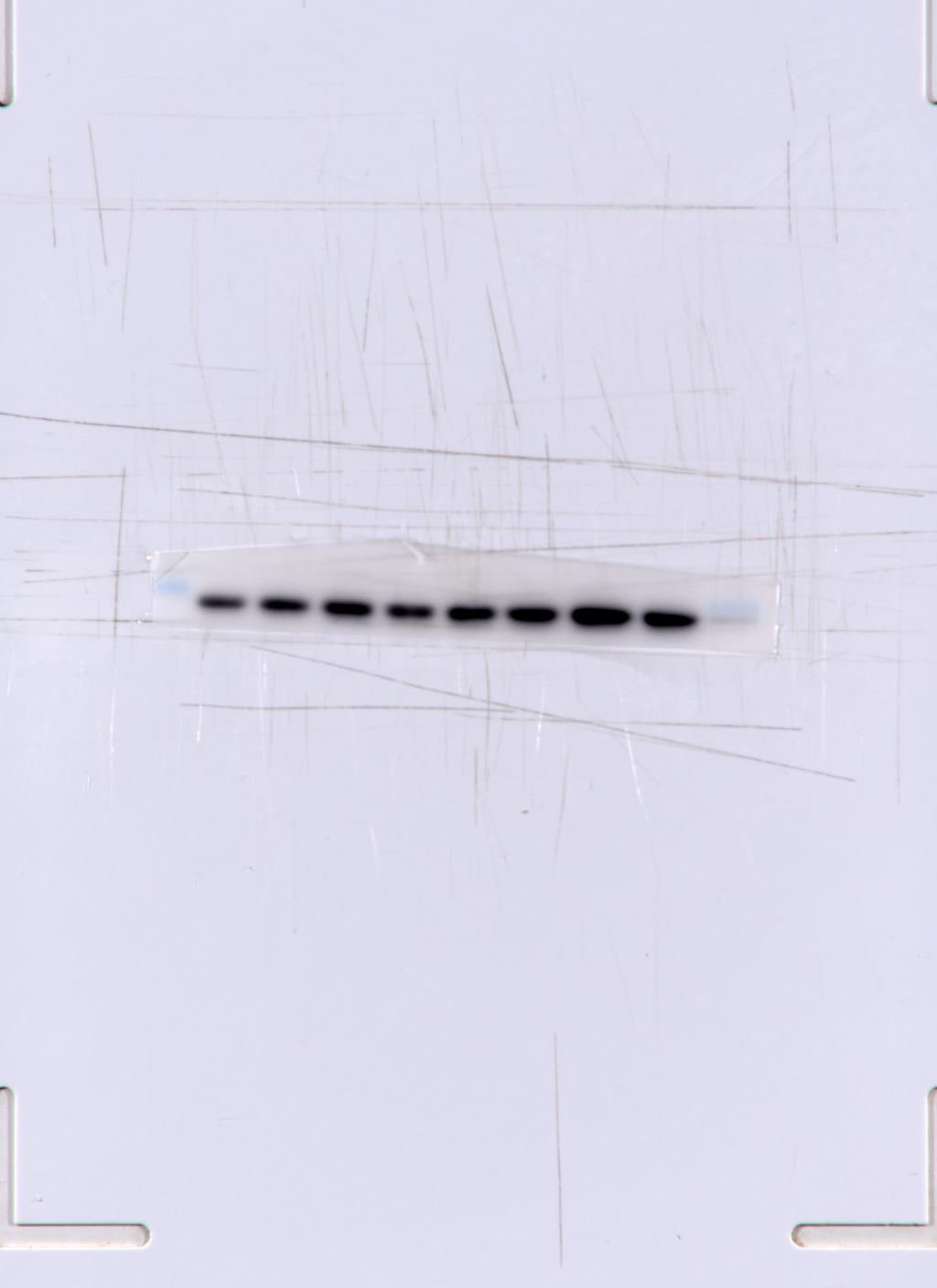

Supplement: Supplementary file 1 [file DataSheet1.ZIP › Supplementary_Material/Fig 4 B, H, J/Fig 4 J/Fig 4 J HO-1.jpg]

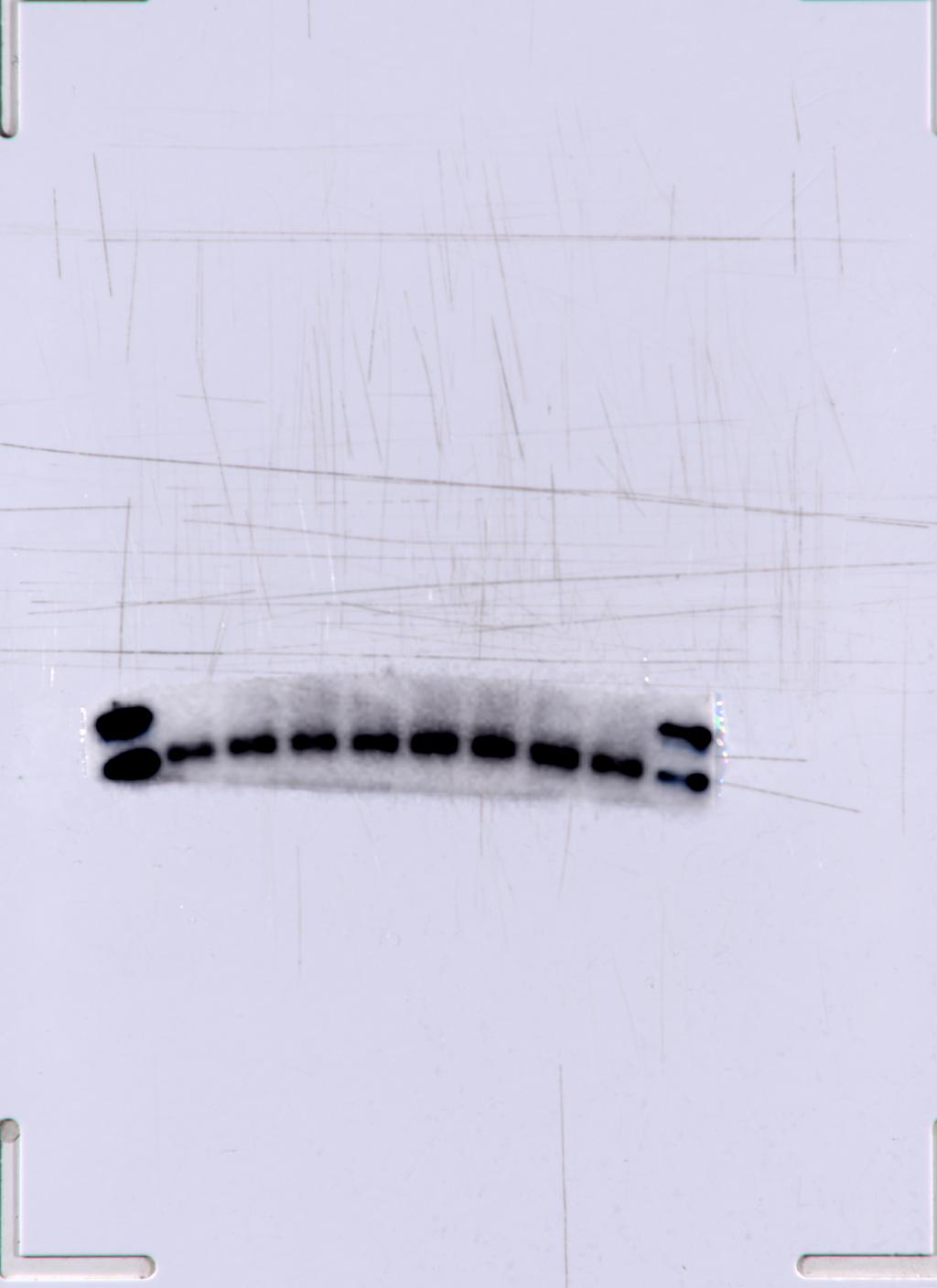

Supplement: Supplementary file 1 [file DataSheet1.ZIP › Supplementary_Material/Fig 4 B, H, J/Fig 4 J/Fig 4 J NRF2 Cyltoplasm.jpg]

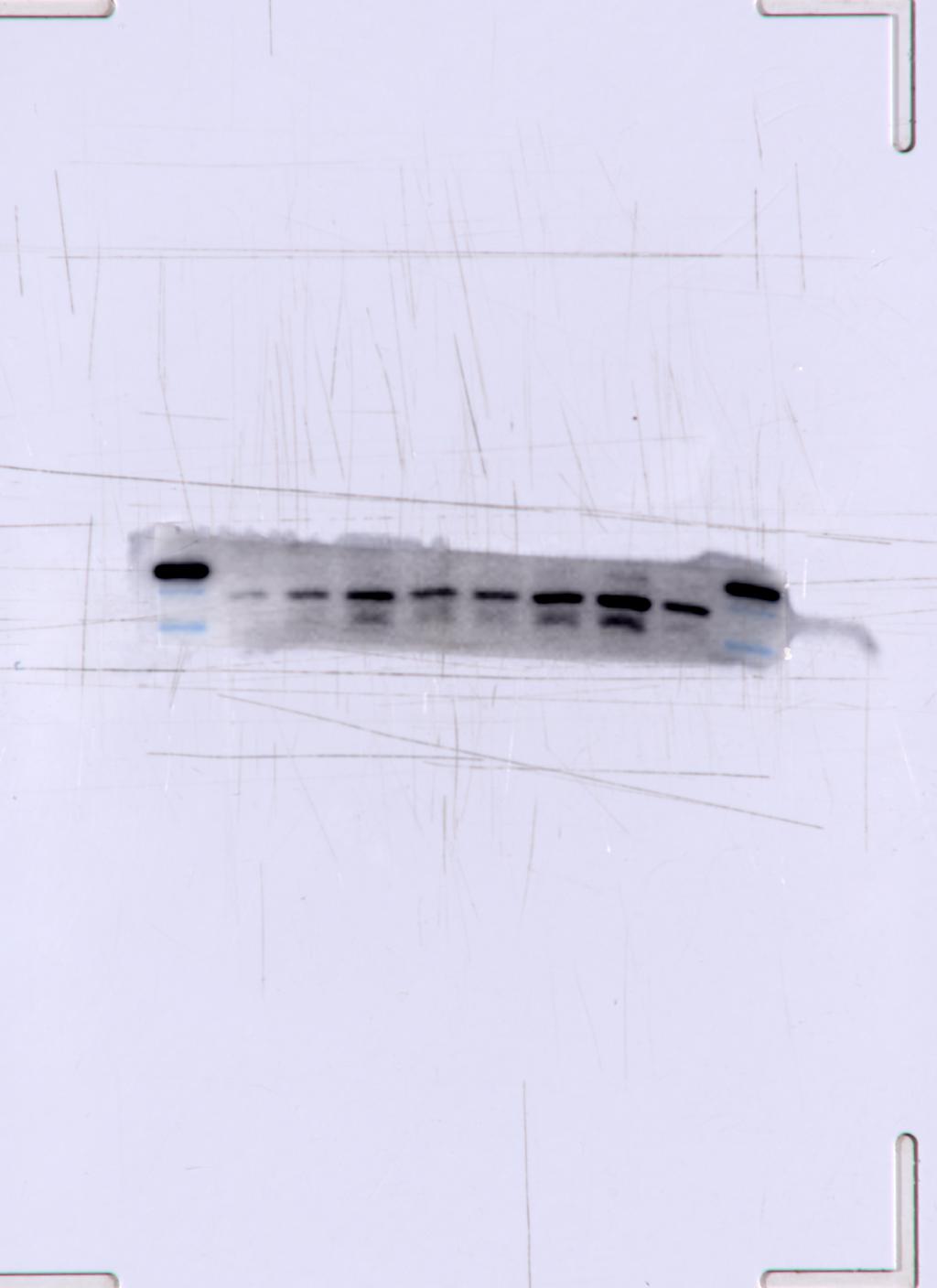

Supplement: Supplementary file 1 [file DataSheet1.ZIP › Supplementary_Material/Fig 4 B, H, J/Fig 4 J/Fig 4 J NRF2 Nucleus.jpg]

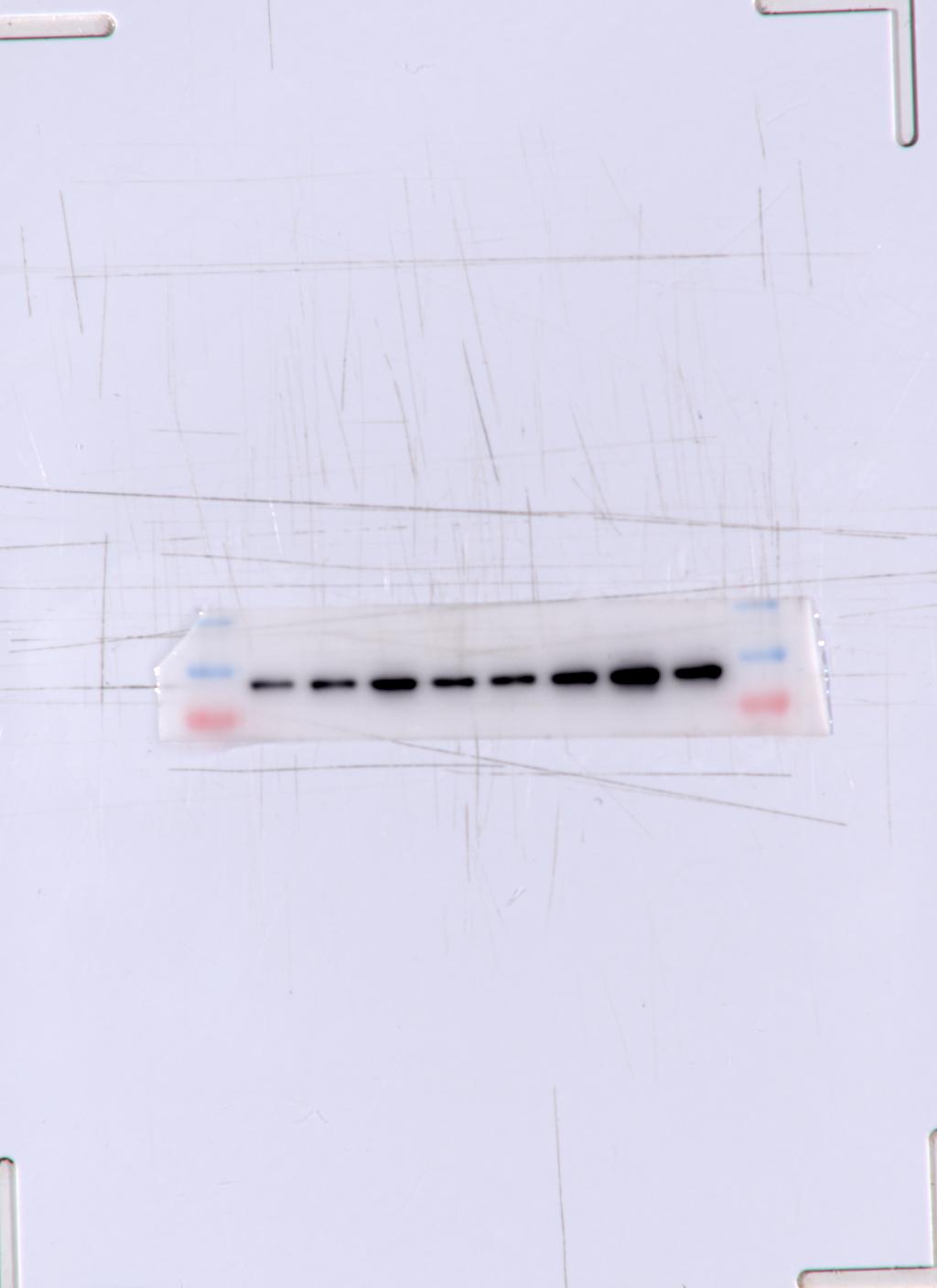

Supplement: Supplementary file 1 [file DataSheet1.ZIP › Supplementary_Material/Fig 4 B, H, J/Fig 4 J/Fig 4 J P-PKC.jpg]

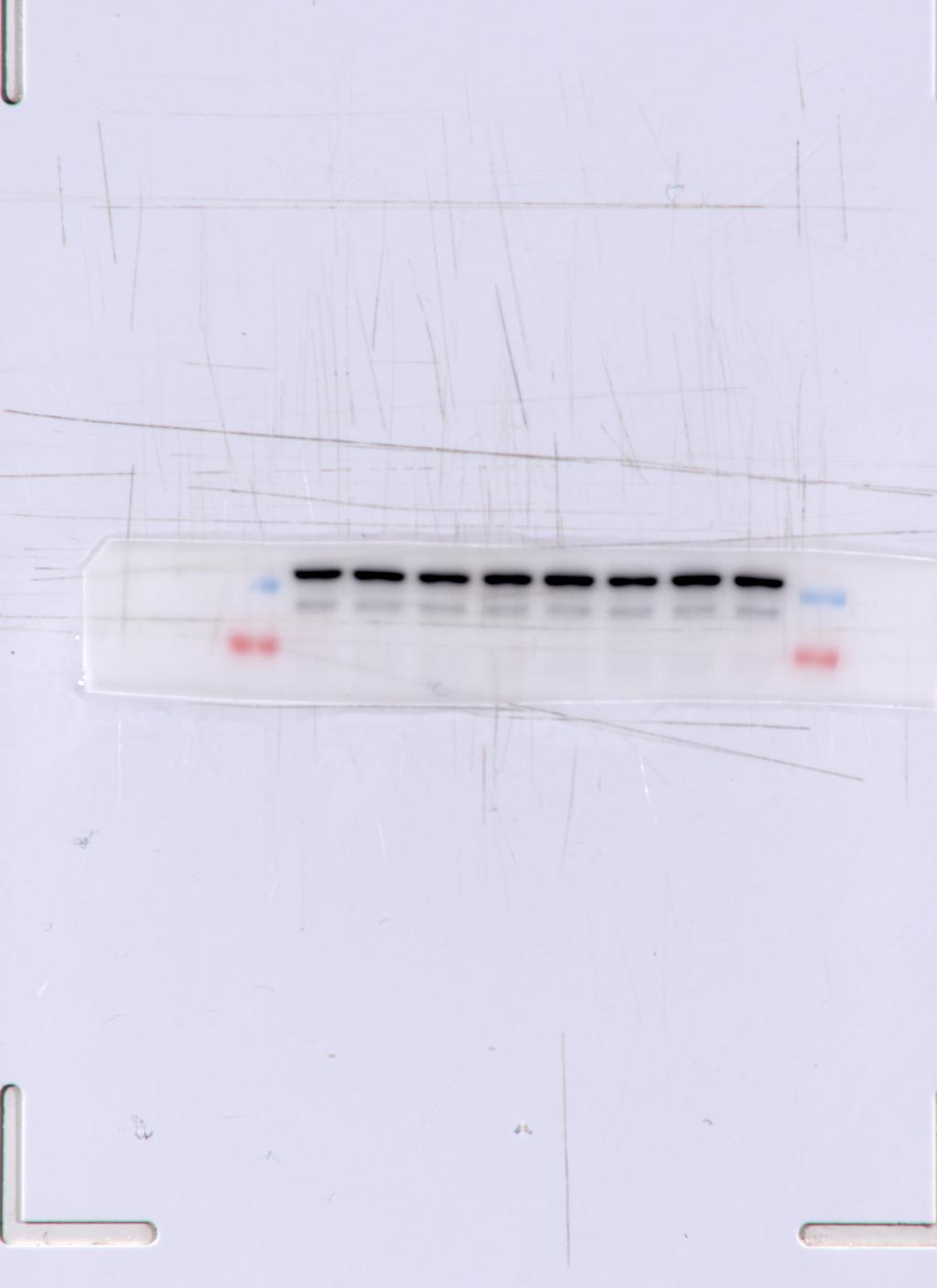

Supplement: Supplementary file 1 [file DataSheet1.ZIP › Supplementary_Material/Fig 4 B, H, J/Fig 4 J/Fig 4 J PKC.jpg]

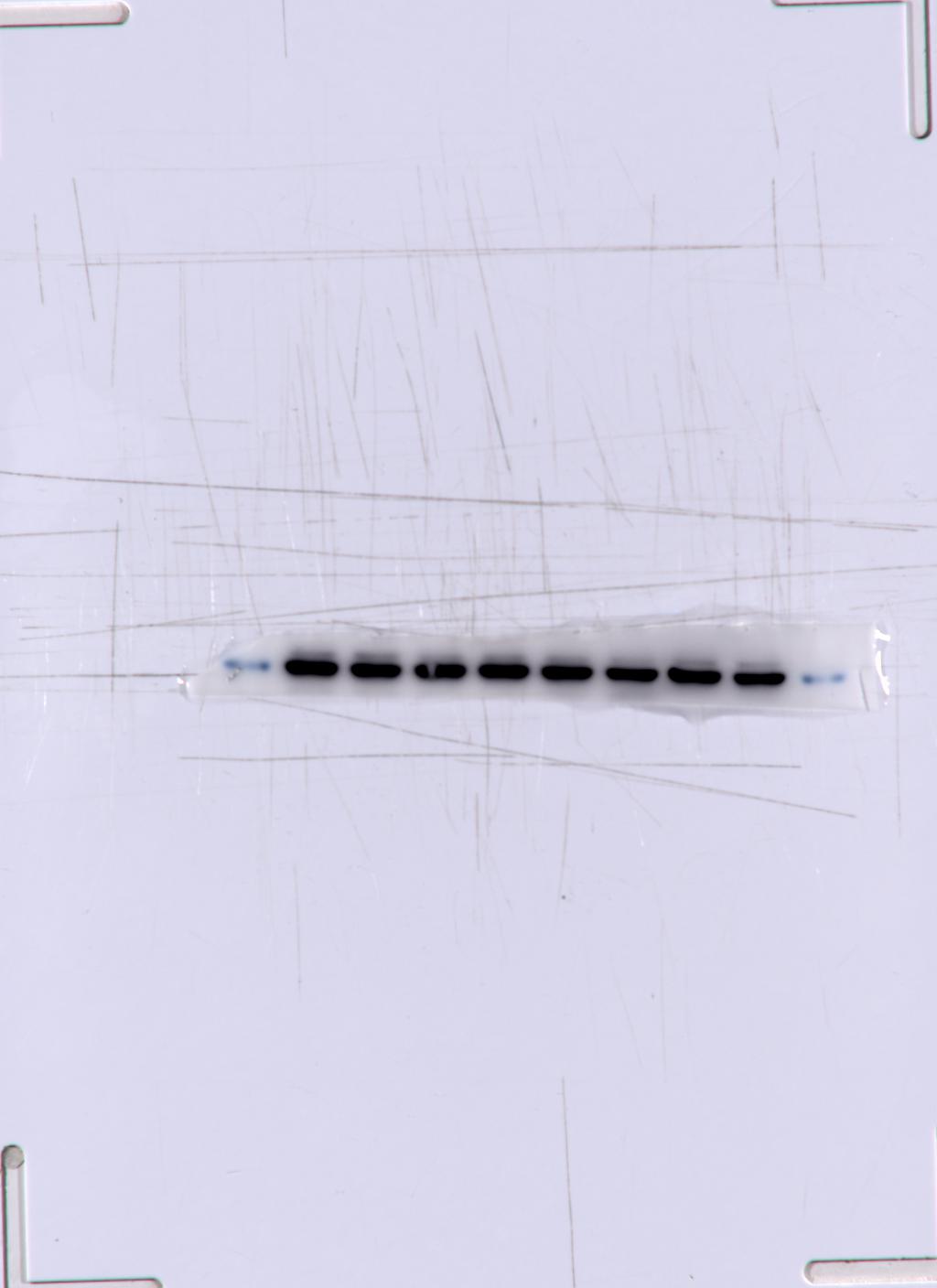

Supplement: Supplementary file 1 [file DataSheet1.ZIP › Supplementary_Material/Fig 4 B, H, J/Fig 4 J/Fig 4 J α-tubulin Cyltoplasm.jpg]

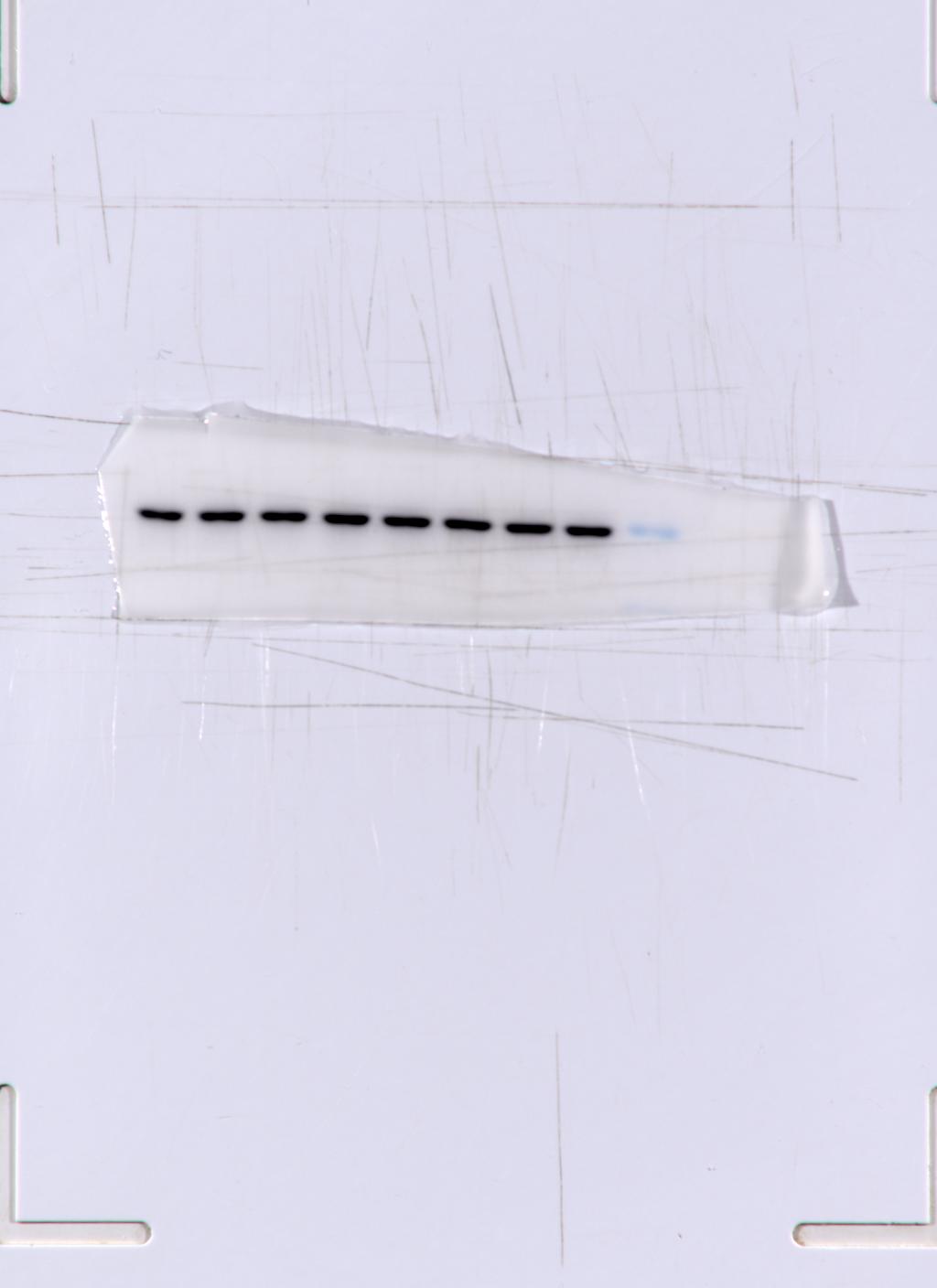

Supplement: Supplementary file 1 [file DataSheet1.ZIP › Supplementary_Material/Fig 4 B, H, J/Fig 4 J/Fig 4 J α-tubulin.jpg]

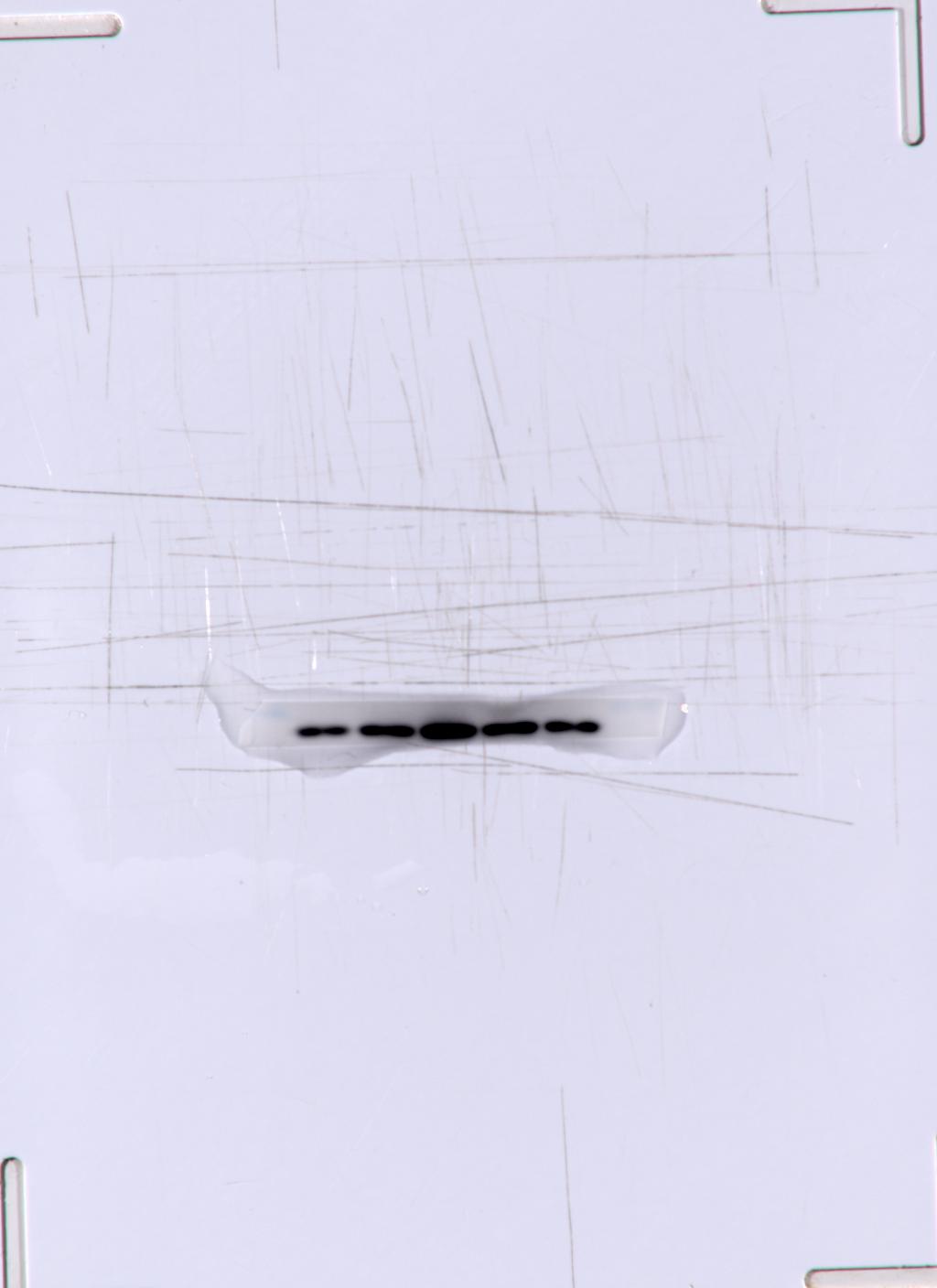

Supplement: Supplementary file 1 [file DataSheet1.ZIP › Supplementary_Material/Fig 5 K/F 5 K HO-1.jpg]

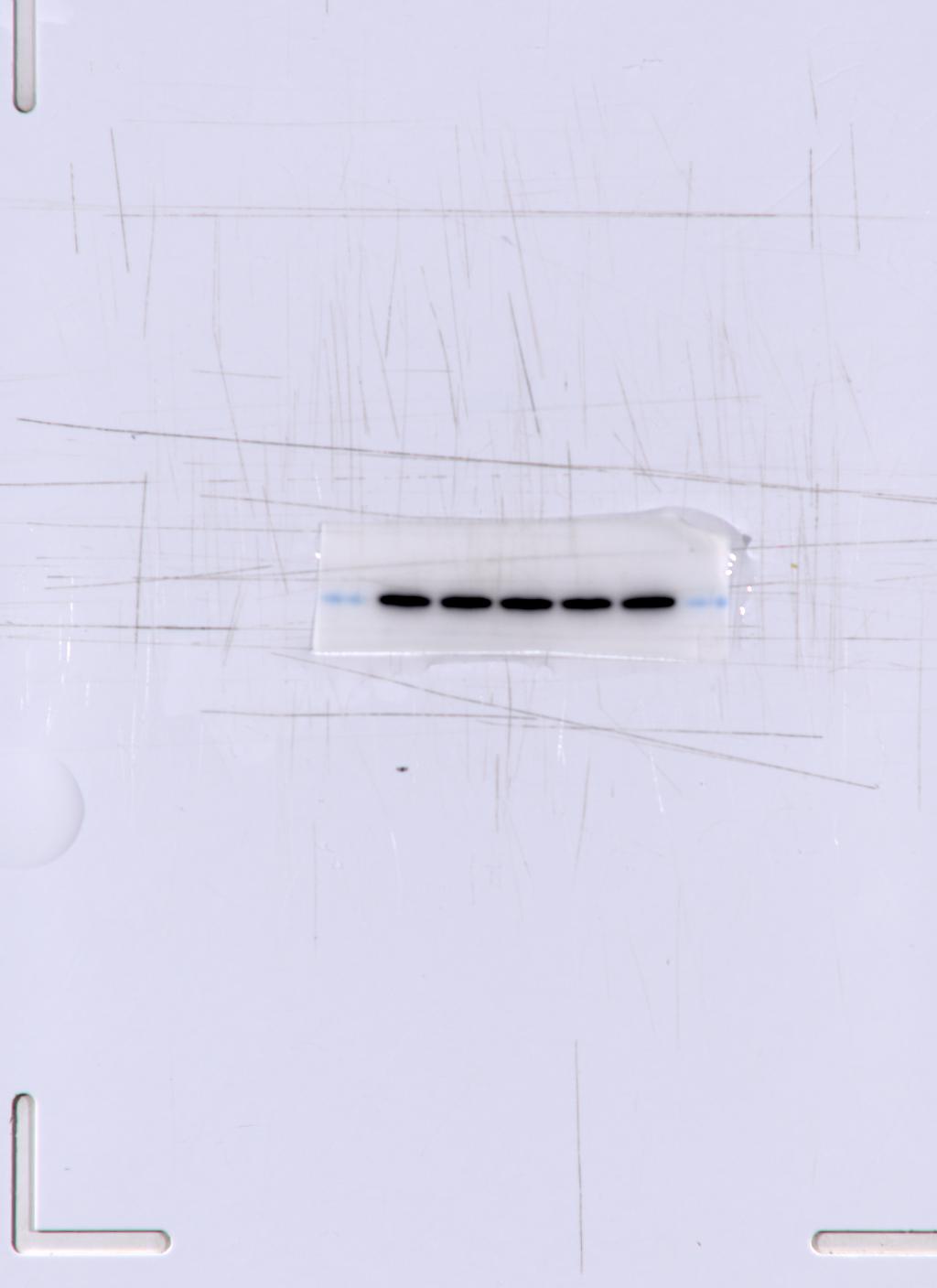

Supplement: Supplementary file 1 [file DataSheet1.ZIP › Supplementary_Material/Fig 5 K/Fig 5 K Histone H3 Nucleus.jpg]

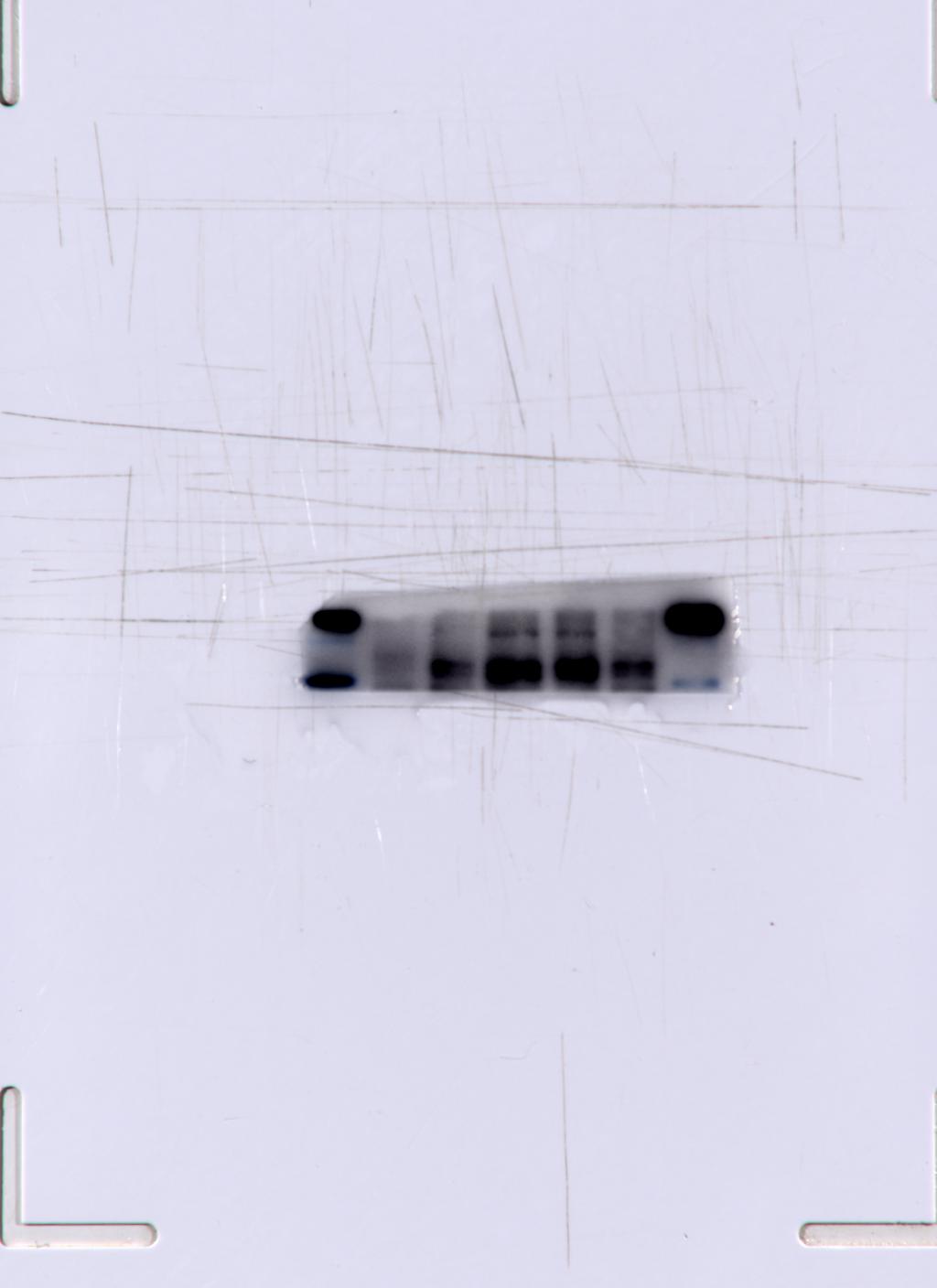

Supplement: Supplementary file 1 [file DataSheet1.ZIP › Supplementary_Material/Fig 5 K/Fig 5 K NRF2 Nucleus.jpg]

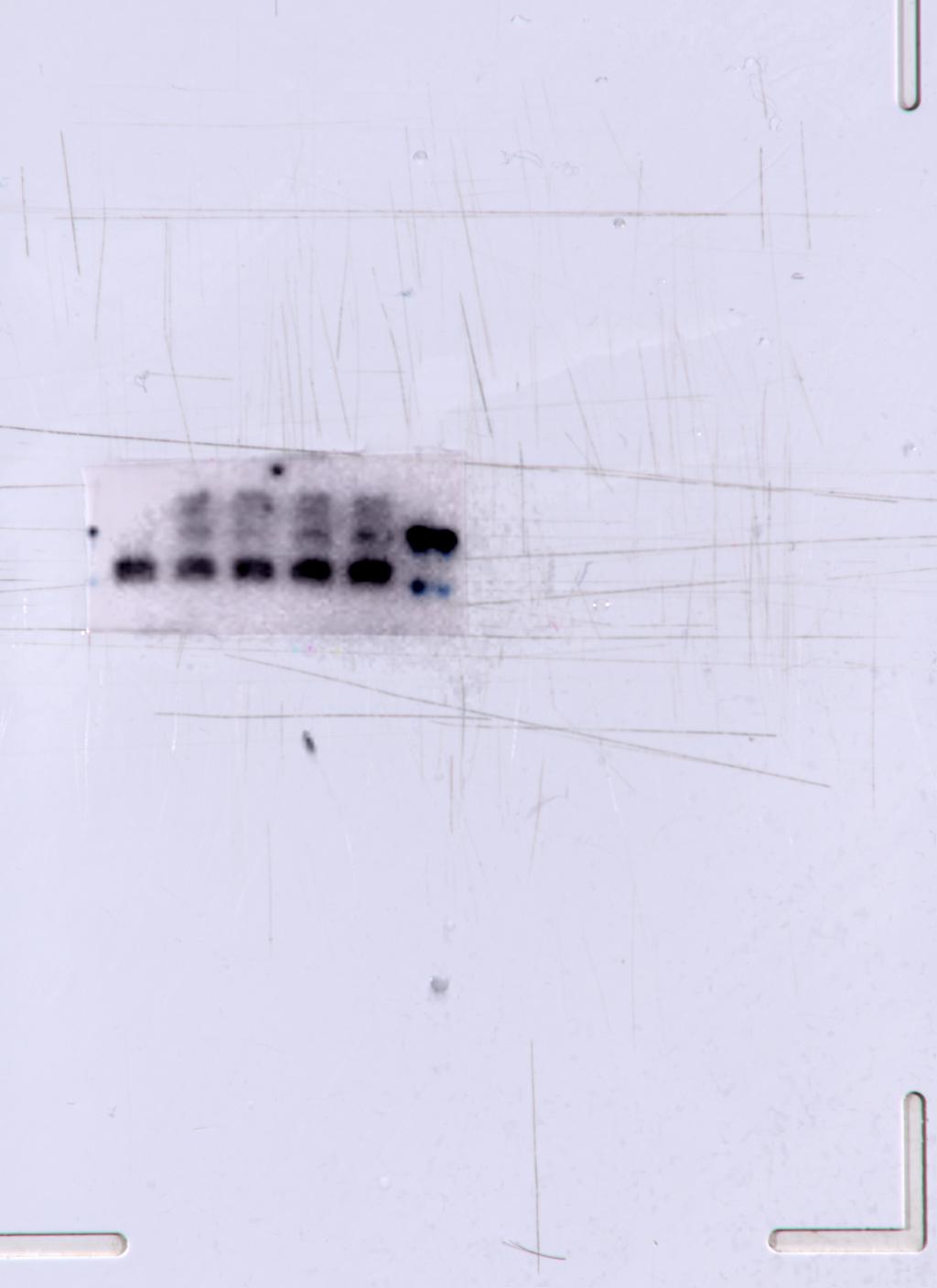

Supplement: Supplementary file 1 [file DataSheet1.ZIP › Supplementary_Material/Fig 5 K/Fig 5 K NRF2 Cytoplasm.jpg]

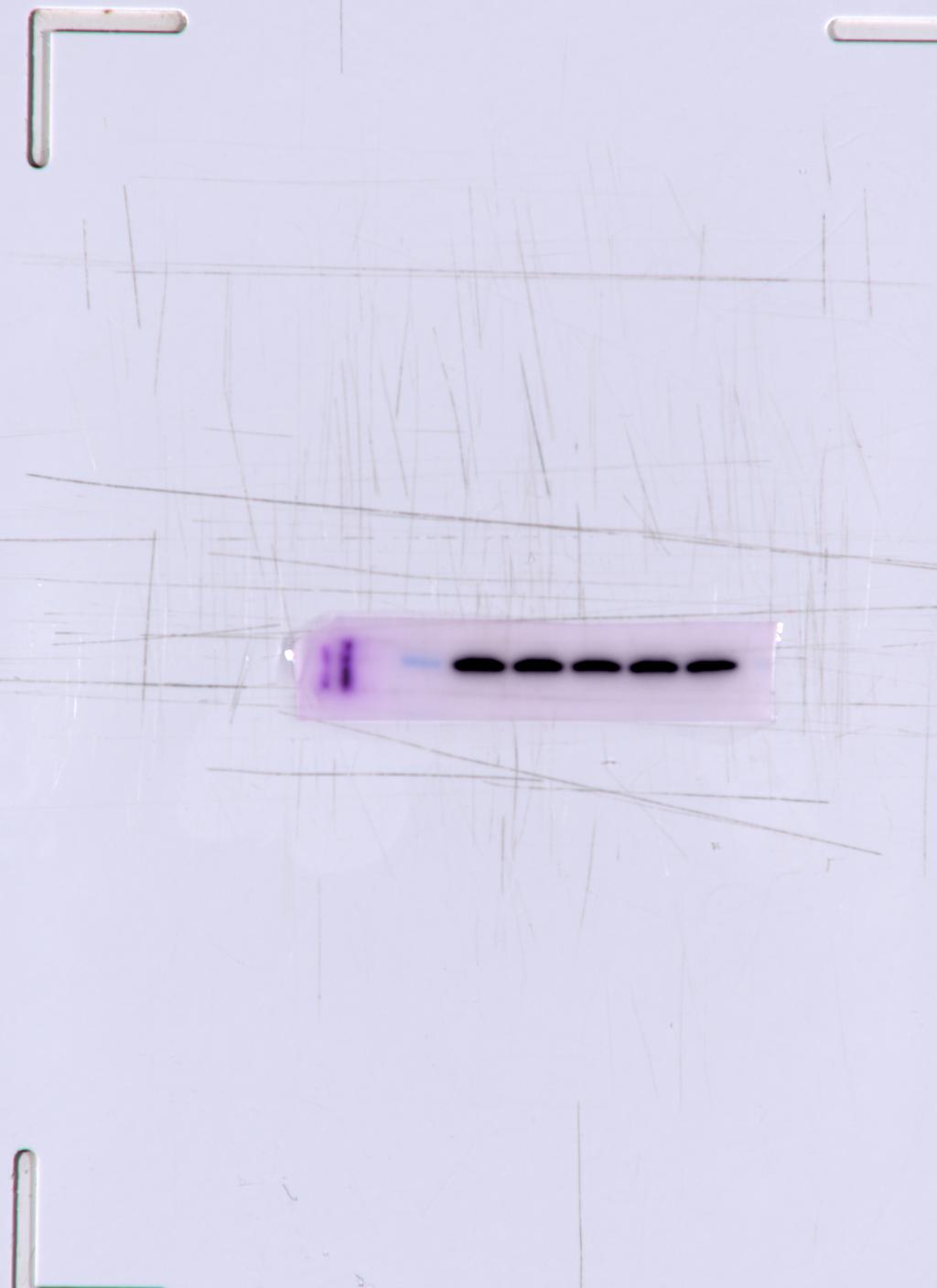

Supplement: Supplementary file 1 [file DataSheet1.ZIP › Supplementary_Material/Fig 5 K/Fig 5 K α-tubulin Cytoplasm.jpg]

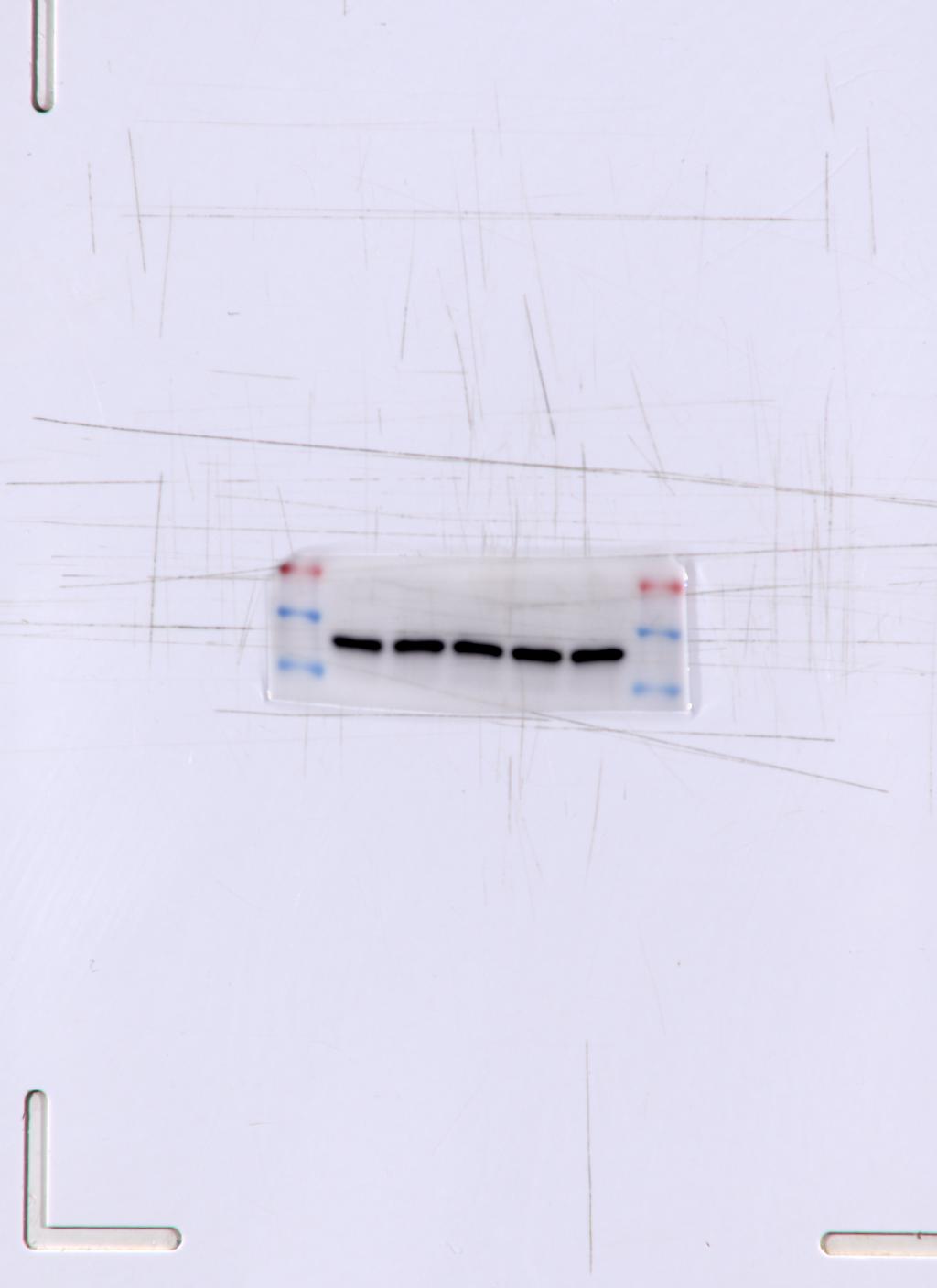

Supplement: Supplementary file 1 [file DataSheet1.ZIP › Supplementary_Material/Fig 5 K/Fig 5 K α-tubulin.jpg]

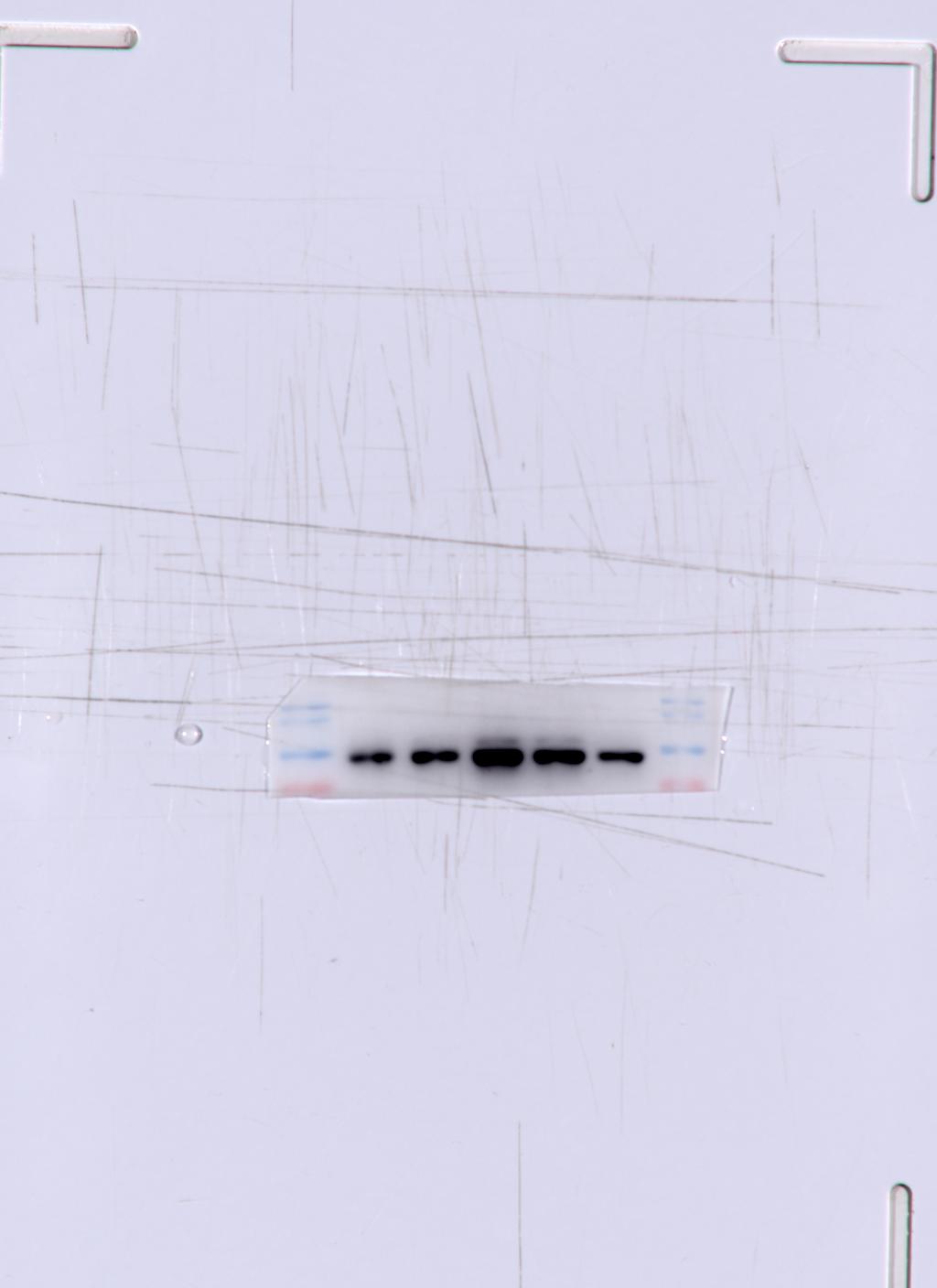

Supplement: Supplementary file 1 [file DataSheet1.ZIP › Supplementary_Material/Fig 6 A/Fig 6 A P-PKC.jpg]

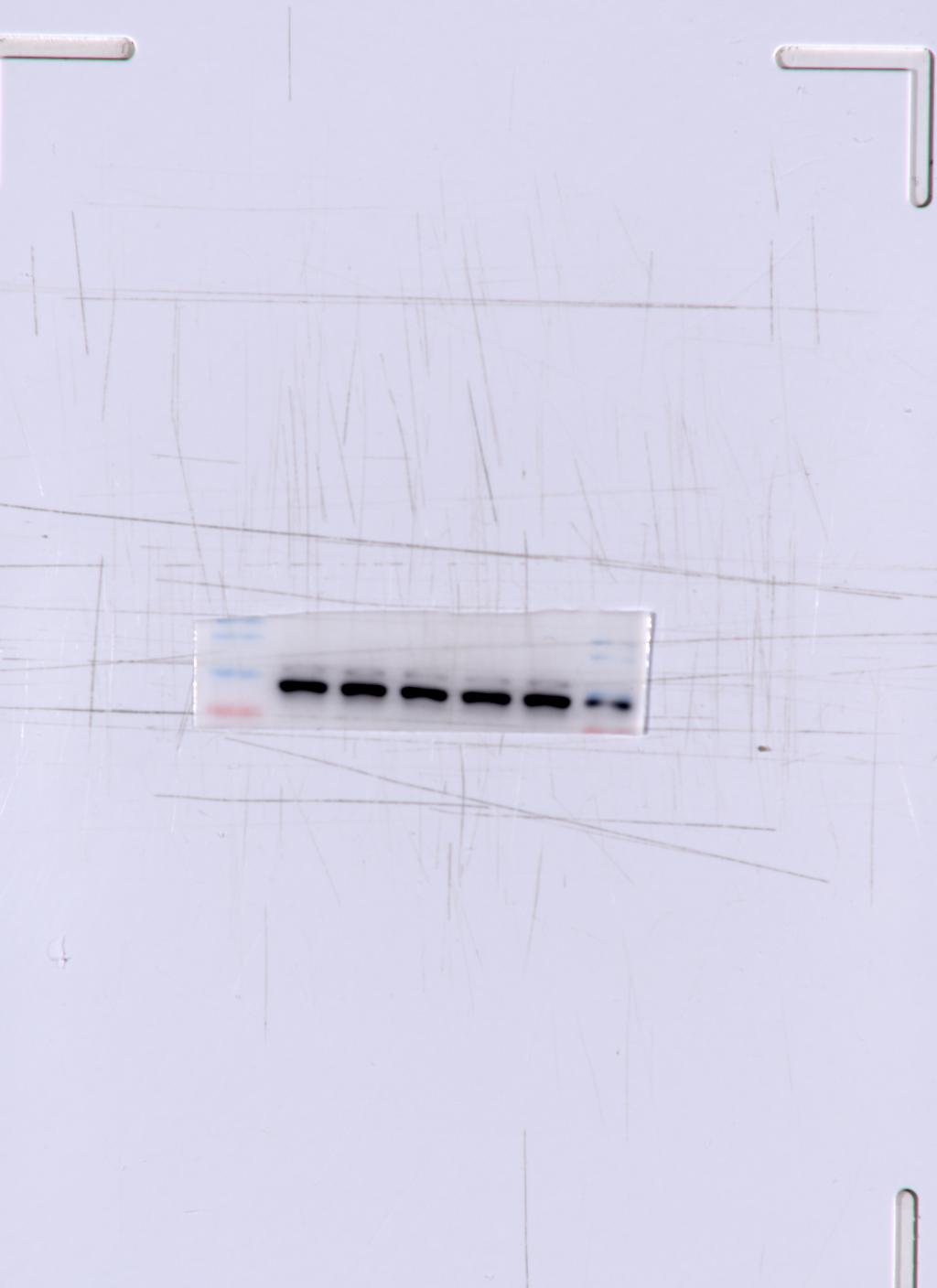

Supplement: Supplementary file 1 [file DataSheet1.ZIP › Supplementary_Material/Fig 6 A/Fig 6 A PKC.jpg]

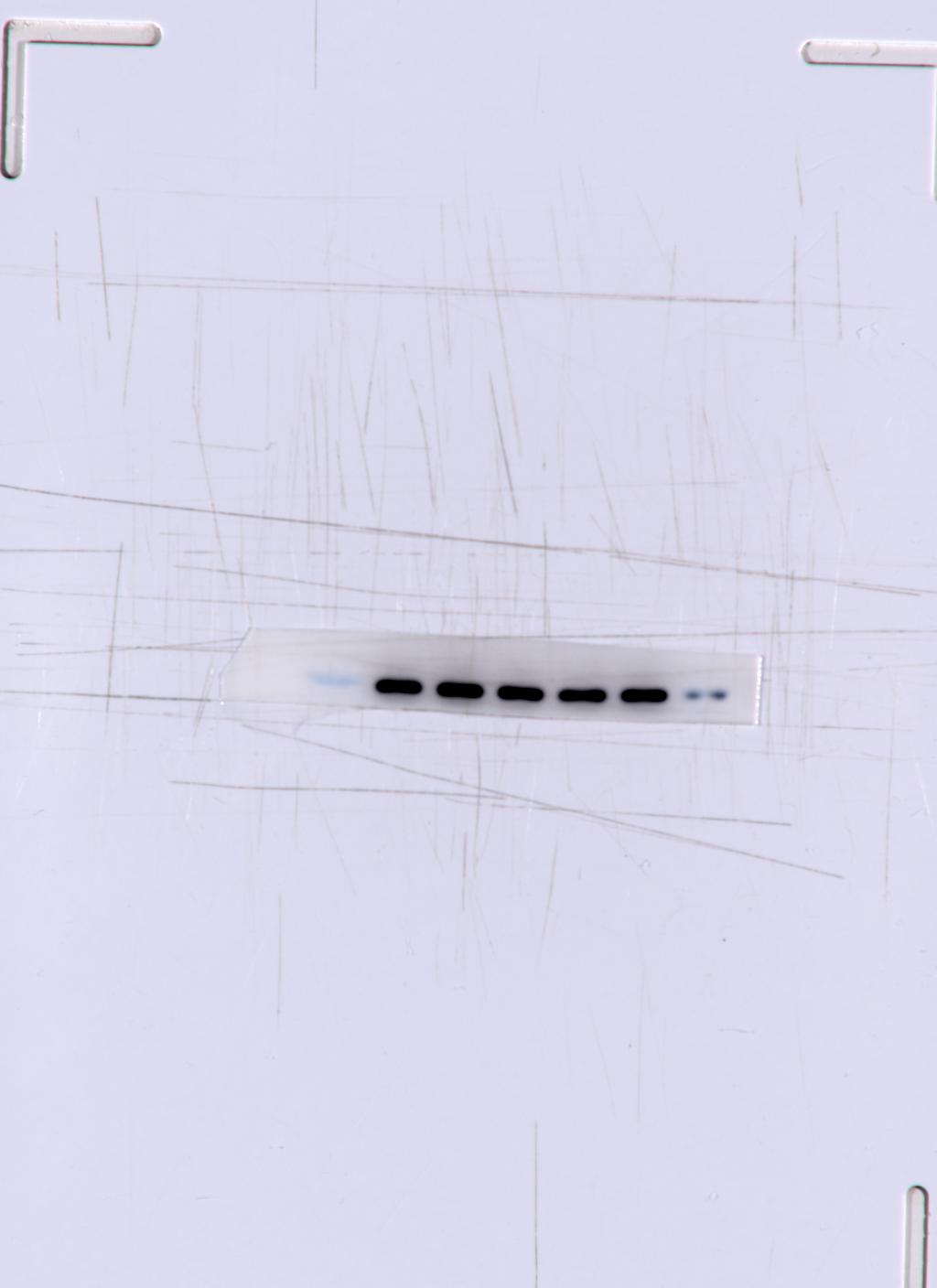

Supplement: Supplementary file 1 [file DataSheet1.ZIP › Supplementary_Material/Fig 6 A/Fig 6 A α-tubulin.jpg]
