# Supplementary material for: Propofol Inhibits Ischemia/Reperfusion-Induced Cardiotoxicity Through the Protein Kinase C/Nuclear Factor Erythroid 2-Related Factor Pathway
Source: Front Pharmacol. 2021 May 13;12:655726. doi: 10.3389/fphar.2021.655726 (PMC8155638; doi:10.3389/fphar.2021.655726)

**Supplemental figure S1**: Full scan of the original blots of cropped images shown in Figure 4 B.

Lane1 and lane4: marker. Lane2: Scramble siRNA. Lane3: PKC siRNA

α-tubulin PKC


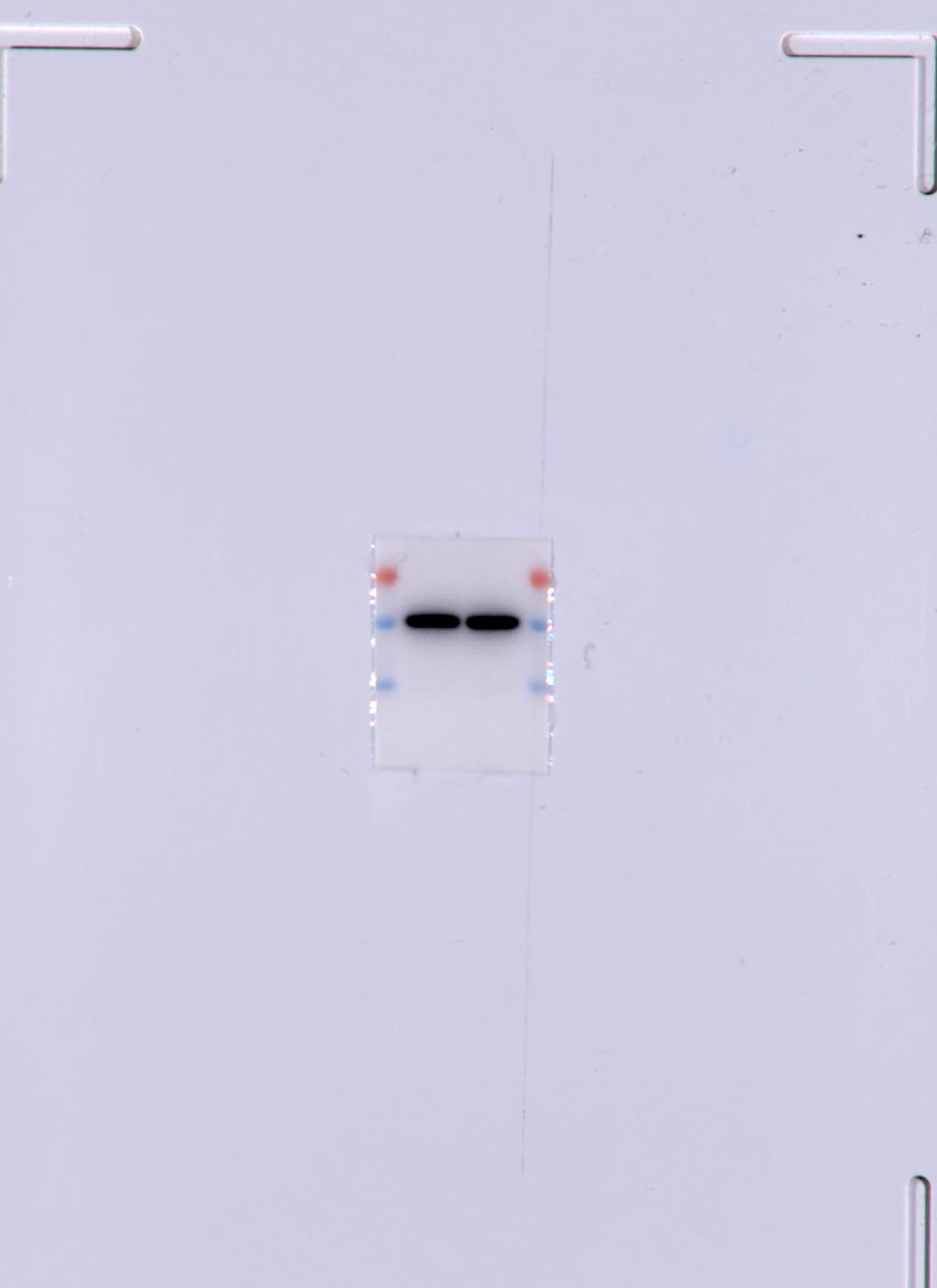

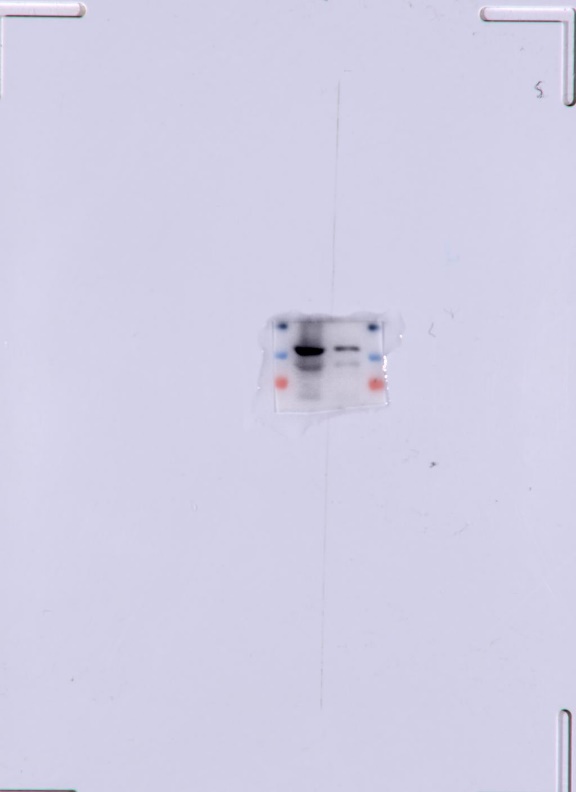


**Supplemental figure S2**: Full scan of the original blots of cropped images shown in Figure 4 H.

Lane1,4,7 lane10: marker. Lane2: Control A. Lane3: Pcontrol A. Lane5: Control B (chosen). Lane6: Pcontrol B (chosen). Lane8: Control C. Lane9: Pcontrol C.

p-pkc pkc


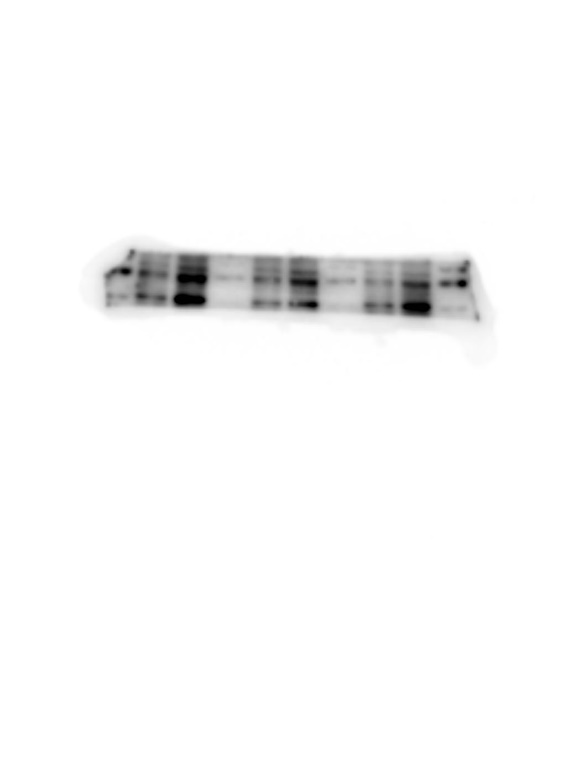

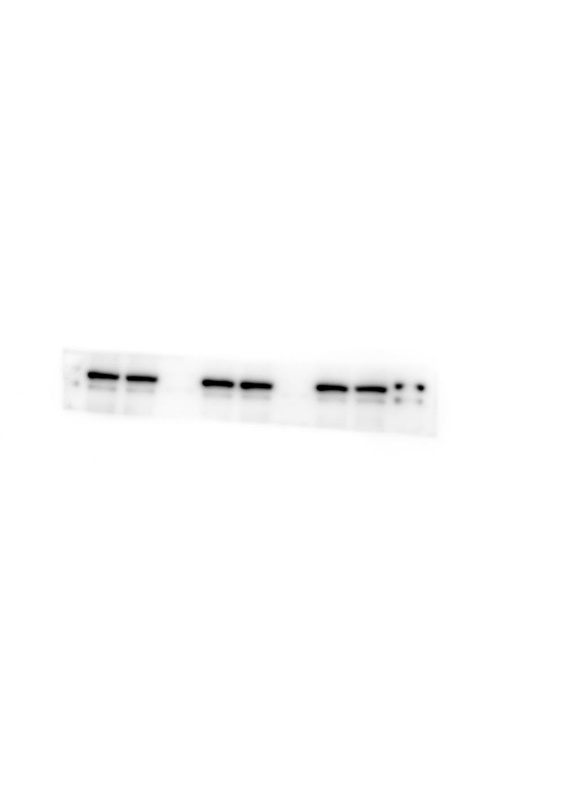


α-tubulin


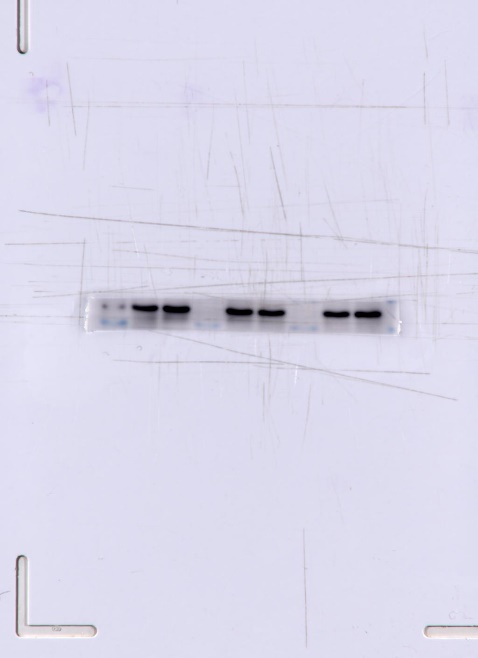


**Supplemental figure S3**: Full scan of the original blots of cropped images shown in Figure 4 J.

Lane1 and 10: marker. Lane2: Control. Lane3: H. Lane4: H+P. Lane5: H+DMSO (deletion). Lane6: Pcontrol. Lane7: PH. Lane8: PH+P. Lane9: PH+DMSO (deletion).

NRF2 Nucleus Histone H3 Nuleus


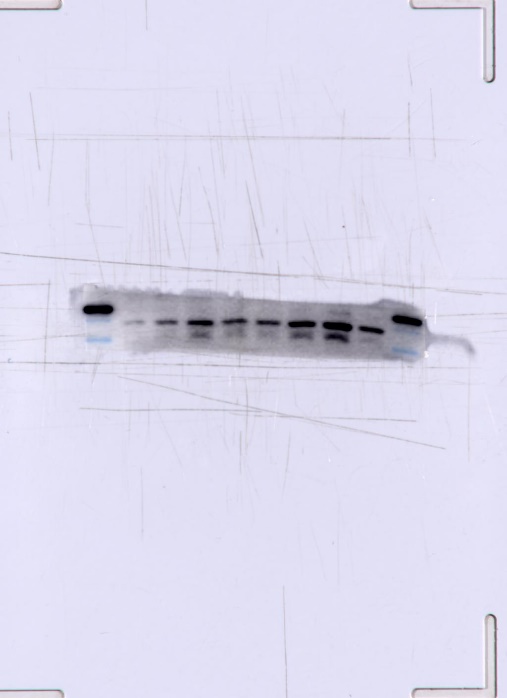

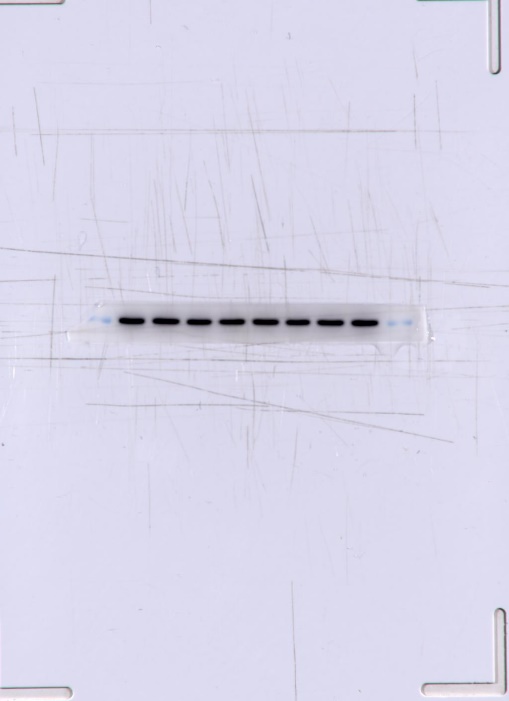


NRF2 Cyltoplasm α-tubulin Cyltoplasm


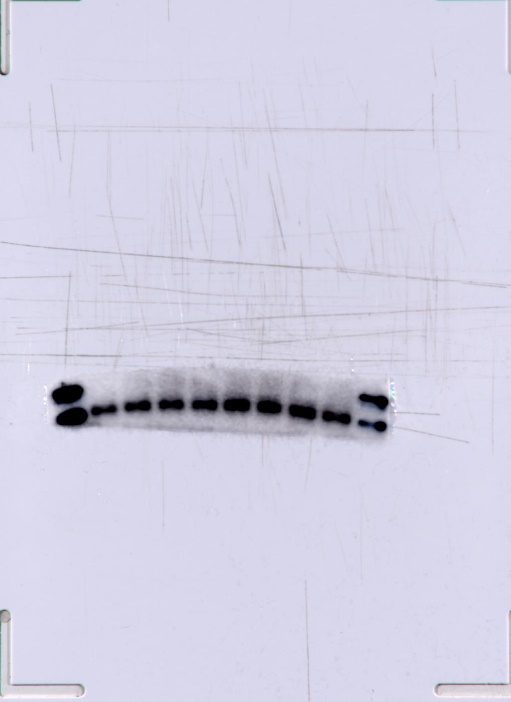

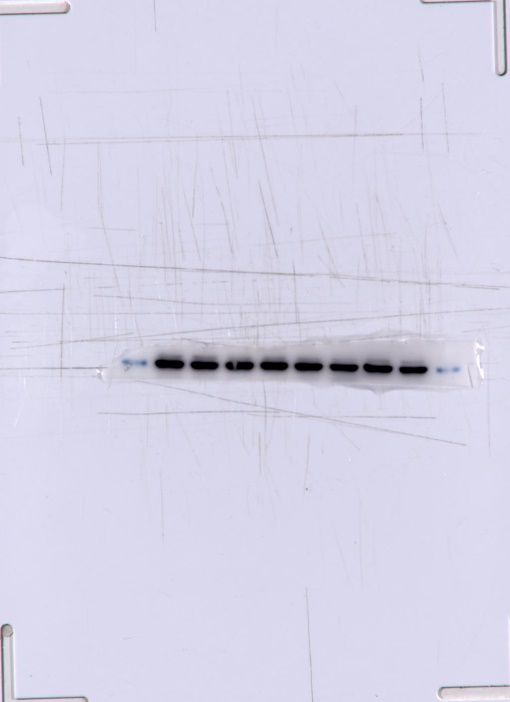


P-PKC PKC


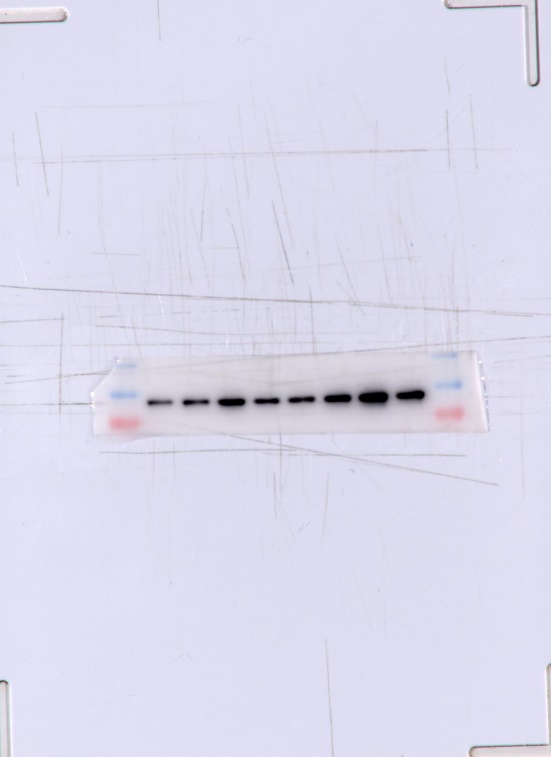

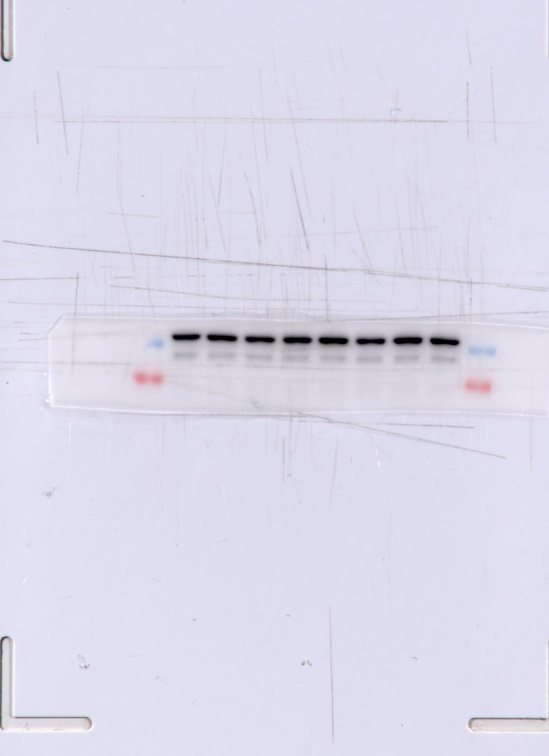


HO-1 α-tubulin


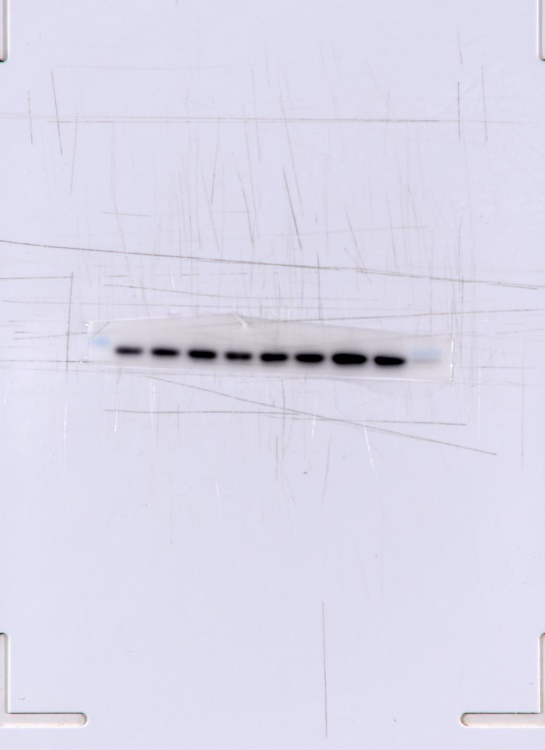

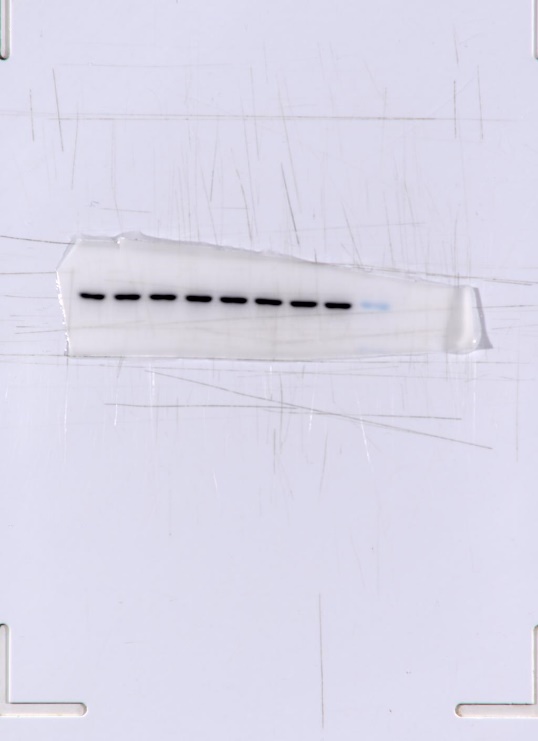


**Supplemental figure S4**: Full scan of the original blots of cropped images shown in Figure 5 K.

Lane1 and lane7: marker. Lane2: Control. Lane3: I/R. Lane4: I/R+P. Lane5:I/R+P+CHE. Lane6: I/R+CHE (deletion).

NRF2 Nucleus Histone H3 Nucleus


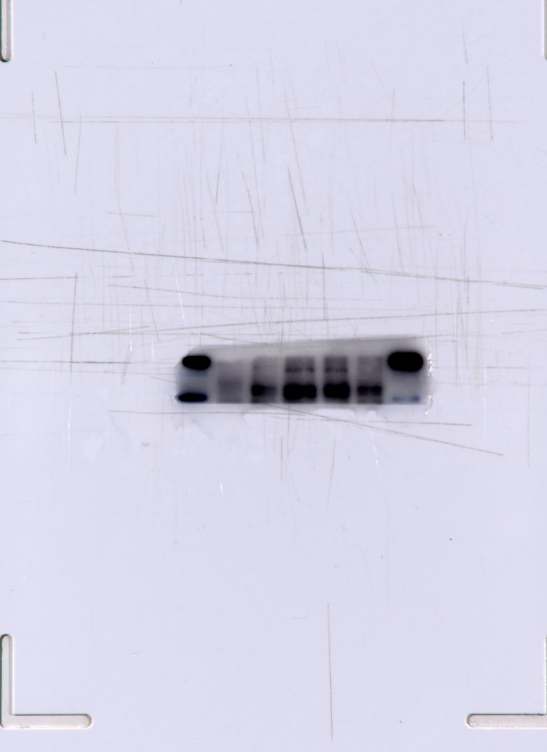

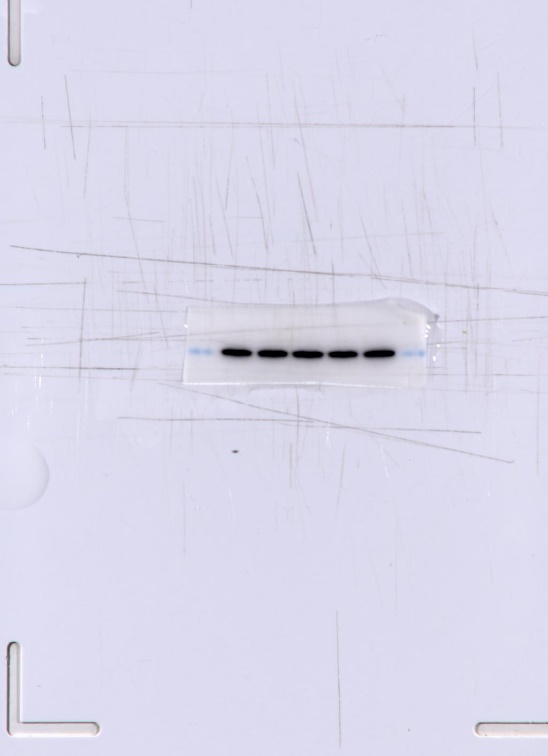


NRF2 Cytoplasm α-tubulin Cytoplasm


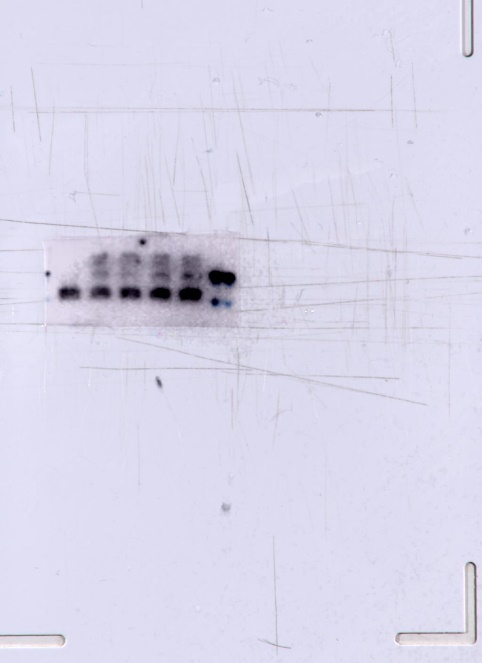

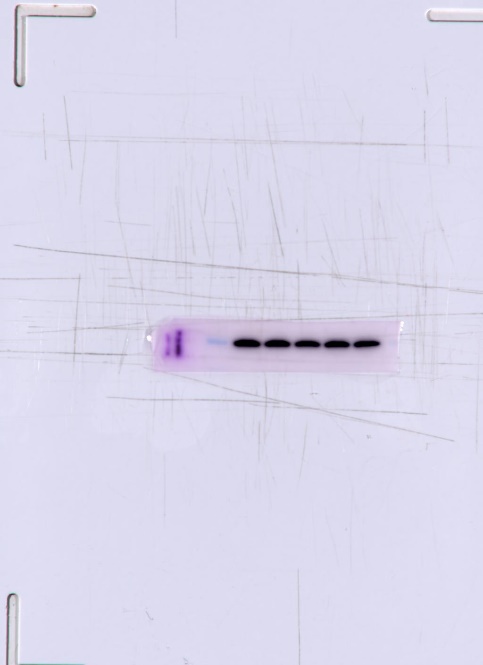


**Supplemental figure S5**: Full scan of the original blots of cropped images shown in Figure 6 A.

Lane1 and lane7: marker. Lane2: Control. Lane3: I/R. Lane4: I/R+P. Lane5: I/R+P+CHE. Lane6: I/R+CHE (deletion).

P-PKC PKC


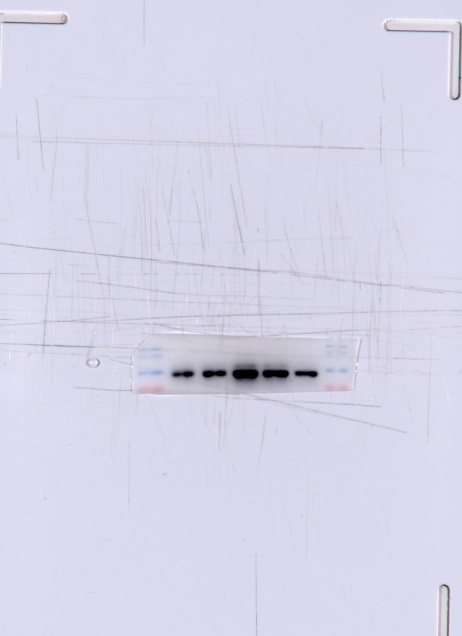

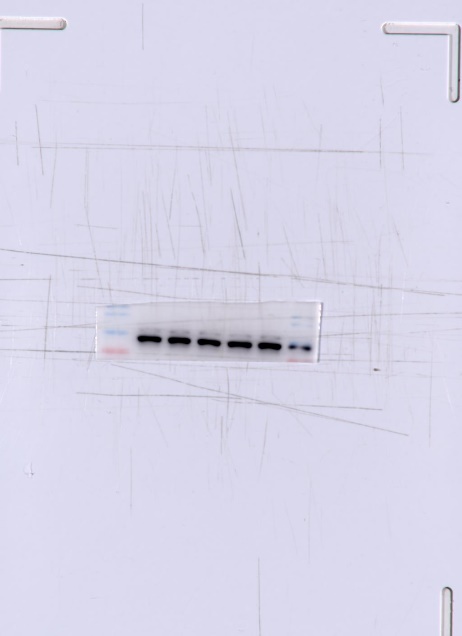


α-tubulin


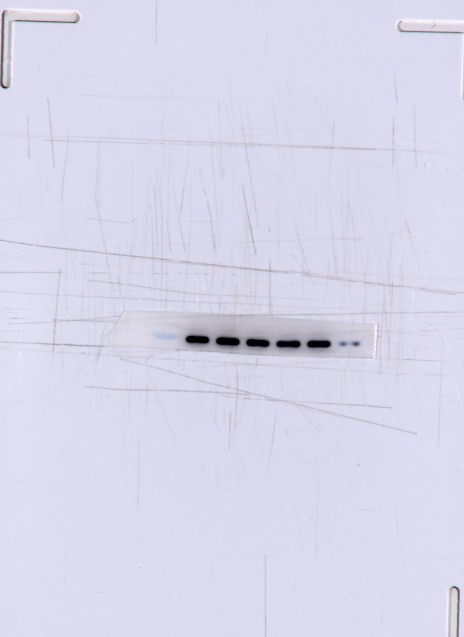

Supplement: Supplementary file 2 [file DataSheet1.docx]
